# BAIAP3, a C2 domain–containing Munc13 protein, controls the fate of dense-core vesicles in neuroendocrine cells

**DOI:** 10.1083/jcb.201702099

**Published:** 2017-07-03

**Authors:** Xingmin Zhang, Shan Jiang, Kelly A. Mitok, Lingjun Li, Alan D. Attie, Thomas F.J. Martin

**Affiliations:** 1 Department of Biochemistry, University of Wisconsin, Madison, WI; 2 School of Pharmacy, University of Wisconsin, Madison, WI; 3 Program in Cellular and Molecular Biology, University of Wisconsin, Madison, WI

## Abstract

Dense-core vesicle (DCV) exocytosis is a SNARE (soluble *N*-ethylmaleimide–sensitive fusion attachment protein receptor)-dependent anterograde trafficking pathway that requires multiple proteins for regulation. Several C2 domain–containing proteins are known to regulate Ca^2+^-dependent DCV exocytosis in neuroendocrine cells. In this study, we identified others by screening all (∼139) human C2 domain–containing proteins by RNA interference in neuroendocrine cells. 40 genes were identified, including several encoding proteins with known roles (CAPS [calcium-dependent activator protein for secretion 1], Munc13-2, RIM1, and SYT10) and many with unknown roles. One of the latter, BAIAP3, is a secretory cell–specific Munc13-4 paralog of unknown function. BAIAP3 knockdown caused accumulation of fusion-incompetent DCVs in BON neuroendocrine cells and lysosomal degradation (crinophagy) of insulin-containing DCVs in INS-1 β cells. BAIAP3 localized to endosomes was required for Golgi trans-Golgi network 46 (TGN46) recycling, exhibited Ca^2+^-stimulated interactions with TGN SNAREs, and underwent Ca^2+^-stimulated TGN recruitment. Thus, unlike other Munc13 proteins, BAIAP3 functions indirectly in DCV exocytosis by affecting DCV maturation through its role in DCV protein recycling. Ca^2+^ rises that stimulate DCV exocytosis may stimulate BAIAP3-dependent retrograde trafficking to maintain DCV protein homeostasis and DCV function.

## Abstract (precis)

Zhang et al. conducted a siRNA screen of C2 domain proteins involved in regulated peptide secretion. One of the hits, a Munc13 family member BAIAP3, was characterized as endosome localized involved in post-exocytic dense-core vesicle protein recycling to the TGN. BAIAP3 knockdown inhibited dense-core vesicle maturation/stability in neuroendocrine/endocrine cells.

## Introduction

Dense-core vesicle (DCV) exocytosis is essential for peptidergic and aminergic signaling in the nervous, endocrine, and immune systems. DCV biogenesis in the TGN, DCV transport across the cytoplasm, and DCV docking/priming and fusion at the plasma membrane use similar mechanisms in different secretory cell types, but many aspects remain incompletely understood. After budding from the TGN, immature DCVs undergo maturation by endosomal retrieval to become fully functional (Kögel and Gerdes, 2010). Mature DCVs are recruited to the plasma membrane where they are docked and primed by several protein factors that assemble vesicle and plasma membrane SNARE proteins into trans-complexes (Rizo and Xu, 2015). The Ca^2+^-triggered fusion of DCVs with the plasma membrane is mediated by Ca^2+^ sensors acting on SNARE complexes and the plasma membrane (Südhof and Rothman, 2009; Jahn and Fasshauer, 2012). Unlike synaptic vesicles that recycle locally, DCVs undergo compensatory endocytosis, which recycles DCV membrane proteins through retrograde trafficking to the TGN (Farquhar, 1983; Bauer et al., 2004).

C2 domain–containing proteins play essential roles at multiple steps in anterograde DCV-mediated trafficking. C2 domains are comprised of eight antiparallel β-strands connected by surface loops (Sutton and Sprang, 1998). Some C2 domains bind phospholipids in a Ca^2+^-dependent manner through negatively charged surface loop residues that form Ca^2+^ binding sites with the membrane (Corbalan-Garcia and Gómez-Fernández, 2014). This enables C2 domain–containing proteins to mediate Ca^2+^-dependent membrane processes such as for Munc13-1/2 (C2B domain) function in vesicle priming or for synaptotagmin function in fusion triggering (Südhof, 2014). Some C2 domains do not bind Ca^2+^ but mediate protein–protein interactions, such as for Munc13-1/2 (C2A domain) and CAPS (calcium-dependent activator protein for secretion 1) function in vesicle docking and priming (Lu et al., 2006; Petrie et al., 2016).

Munc13 proteins are an important class of C2 domain–containing proteins, with Munc13-1, -2, and -3 regulating Ca^2+^-dependent vesicle priming in neurons and endocrine cells (Koch et al., 2000; Kwan et al., 2006; Man et al., 2015). Similarly, Munc13-4 is essential for the Ca^2+^-dependent priming of secretory granules in immune cells (Feldmann et al., 2003; Boswell et al., 2012; Johnson et al., 2016) but also functions in the Ca^2+^-dependent regulation of late endosomal fusion events (Ménager et al., 2007; He et al., 2016; Woo et al., 2017). The fifth and most recently discovered Munc13 protein BAIAP3 is expressed in the central nervous system and neuroendocrine cells (Wojcik et al., 2013; Man et al., 2015). It is similar to Munc13-4 (46% amino acid similarity), with N- and C-terminal C2 domains predicted to bind Ca^2+^. However, the cellular role of BAIAP3 and the Ca^2+^-regulated membrane fusion process in which it may function have not been determined.

There are ∼139 genes encoding C2 domain–containing proteins in the human genome. The majority of these have not been studied for a role in regulated DCV exocytosis. We conducted a high throughput siRNA screen for C2 domain–containing proteins involved in regulated DCV exocytosis in neuroendocrine cells. Of the 40 siRNA pools that inhibited Ca^2+^-triggered secretion, the screen identified several well-characterized proteins known to function in DCV exocytosis as well as many proteins of unknown function. The role of the novel Munc13 protein BAIAP3 was fully characterized as a C2-domain protein that controls the activity of DCVs in neuroendocrine and endocrine cells. BAIAP3 was found to operate by localizing to endosomes and regulating DCV protein recycling at the TGN. We propose that, in stimulated cells, Ca^2+^-bound BAIAP3 accelerates the retrograde trafficking of DCV proteins to the TGN to maintain DCV protein balance during the Ca^2+^-stimulated anterograde trafficking of DCVs.

## Results

### C2-domain proteins required for DCV exocytosis

**Figure 1.**
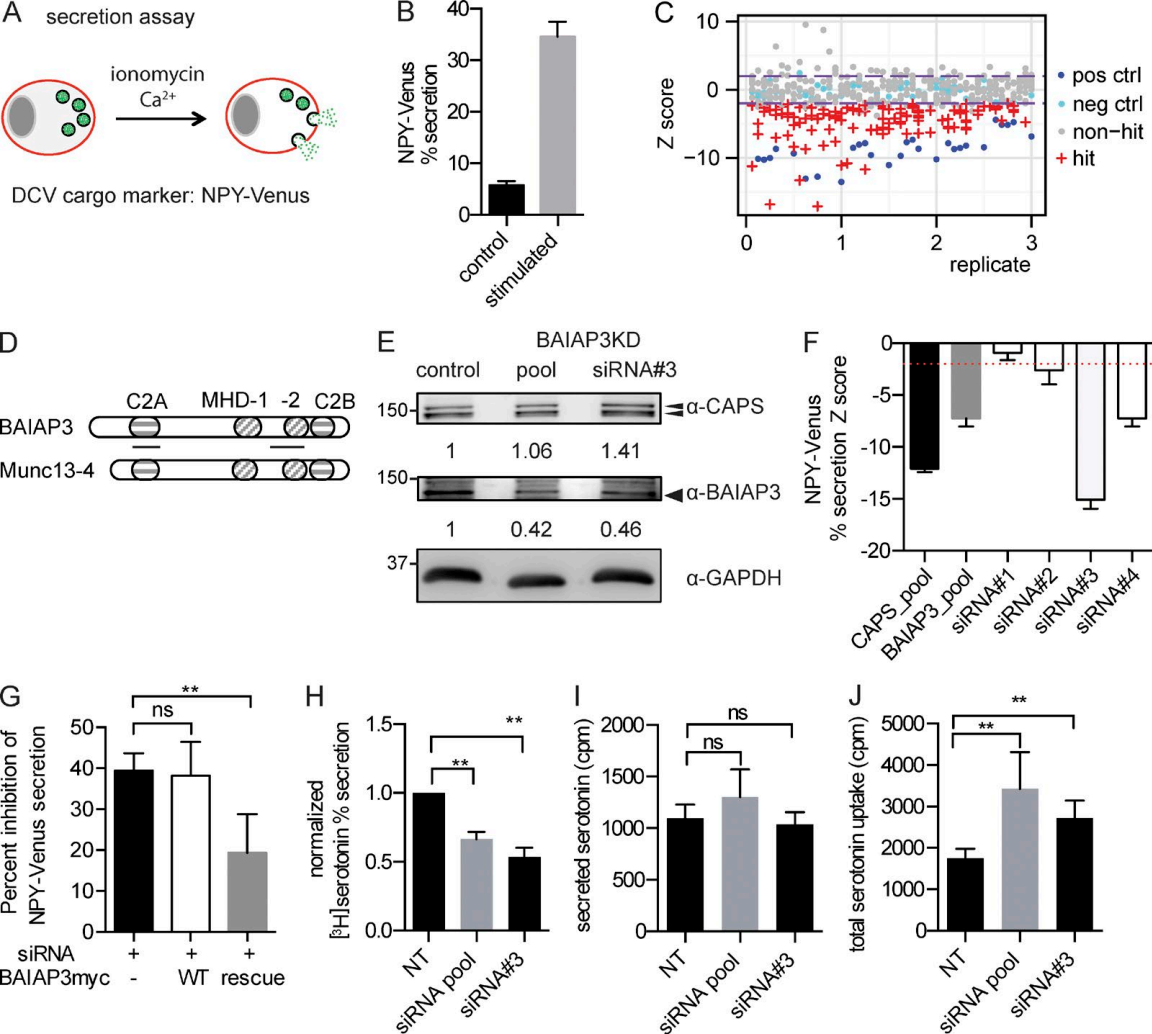
**BAIAP3 is required for acutely stimulated secretion.** (A) Scheme of NPY-Venus secretion assay. (B) NPY-Venus percent secretion for 1.25-µM ionomycin stimulation or DMSO (control) treatment for 10 min in the presence of external 2.2 mM Ca^2+^. *n* = 48 and 45. (C) Triplicated siRNA screen of C2 domain–containing proteins. Each point corresponds to NPY-Venus percent secretion from 1 well after siRNA treatment. Positive control (pos ctrl; blue): *CADPS* siRNA; negative control (neg ctrl; cyan): a nontargeting siRNA; hits (red); non-hits (gray); purple lines: threshold for hit identification (z score = ±2). (D) Alignment of BAIAP3 with Munc13-4. Lines under BAIAP3 indicate esiRNA target regions. Other symbols show the indicated domains. (E) Expression of BAIAP3 in BON cells. The bands are quantified after normalizing to GAPDH. Molecular mass is shown in kilodaltons. (F) Validation of BAIAP3 knockdown effect on NPY-Venus percent secretion with individual siRNA duplexes. *n* = 5. The red dotted line indicates z score = −2. (G) Rescue of NPY-Venus secretion by siRNA-resistant *BAIAP3*. The effect of each treatment is represented as percent inhibition of NPY-Venus secretion. *n* = 5. Parallel studies indicated that ∼43% of the cells were transduced with siRNA-resistant *BAIAP3* accounting for incomplete rescue. (H) Ca^2+^-induced [^3^H]serotonin percent secretion. Nontargeting (NT) siRNA is set as 1. *n* = 4. (I and J) Secreted and total uptake of [^3^H]serotonin (secreted + lysate). *n* = 4. See also Fig. S1. Data are expressed as mean ± SD. P-values were obtained by a two-tailed Student’s *t* test. **, P < 0.01.

Pancreatic neuroendocrine BON cells contain numerous DCVs and exhibit Ca^2+^-triggered release of peptides (neurotensin, pancreastatin, and chromogranins), biogenic amines, or an expressed neuropeptide Y (NPY)–GFP (Parekh et al., 1994; Karatekin et al., 2008). We established an assay with a BON cell line that stably expresses NPY-Venus as a DCV cargo protein (Fig. 1 A). The percent secretion (34.6 ± 0.4%) of cellular NPY-Venus in response to Ca^2+^ influx (ionomycin stimulation) over a basal level (5.7 ± 0.1%) was used to measure acutely stimulated DCV exocytosis (Fig. 1 B). We performed a triplicated siRNA screen of all human C2 domain–containing proteins (Table S1). Z score, which measures the number of SDs from a sample siRNA to the nontargeting siRNA (Birmingham et al., 2009), was used to identify inhibitory siRNAs. siRNA pools targeting three genes required for regulated exocytosis (*CADPS* [CAPS], *SNAP25*, and *STX1A* [syntaxin 1A]) were used as controls. siRNAs targeting *CADPS* strongly inhibited the percent secretion of NPY-Venus (z score = −8.7), whereas targeting *SNAP25* and *STX1A* resulted in milder inhibition (z score = −2.4 and −2.1, respectively). Therefore, we set a z score = −2 as the threshold for hit identification. 40 inhibitory siRNA pools were identified with this threshold (Fig. 1 C and Table S2). Of these, multiple genes with characterized roles in regulated exocytosis, such as *CADPS* (Walent et al., 1992; Ann et al., 1997), *UNC13B* (Varoqueaux et al., 2002), *RIM1/3* (Deng et al., 2011), *PLA2* (Brown et al., 2003), *DOC2A* (Yao et al., 2011), and *Synaptotagmin 10* (*SYT10*; Cao et al., 2011), were identified (Fig. S1 B), which validated the screen. Additional assays were conducted to assess the quality of the screen. For example, *UNC13A* siRNAs did not inhibit NPY-Venus secretion in pilot experiments, and Munc13-4 (*UNC13D*) is not expressed in BON cells (Fig. S1 A). In accord with this, neither gene was identified as a hit, whereas *UNC13B* (Munc13-2) was identified as a significant hit (Fig. S1 B). All *vesicle-associated membrane proteins* (*VAMPs*) were included in the screen as controls. VAMP2 is not expressed in BON cells (Fig. S1 A), and the screen did not identify *VAMP2* as a hit but did identify *VAMP1*, *3*, and *8* as hits (Fig. S1 B). The numerous controls, combined with the identification of known C2 domain–containing proteins for regulated DCV exocytosis, indicated the robustness of the screen.

### BAIAP3 is required for regulated protein secretion

The screen identified *BAIAP3* as a novel gene required for regulated protein secretion. *BAIAP3* encodes a protein originally identified in a yeast two-hybrid assay as a brain-specific angiogenesis inhibitor 1 (BAI1)–interacting protein (Shiratsuchi et al., 1998). BAIAP3 is a Munc13 protein comprised of two Munc13 homology domains (MHDs; Koch et al., 2000) bracketed by two C2 domains predicted to bind Ca^2+^ (Fig. 1 D and Fig. S6) and exhibits >70% sequence similarity to Munc13-4 in MHD and the C2 domain. *BAIAP3* knockout mice display increased seizure propensity and anxiety behavior (Wojcik et al., 2013), which could result from abnormal neuropeptide or serotonin secretion (Kovac and Walker, 2013). A BAIAP3 missense mutation was detected in a search for hypothalamic signaling genes related to extreme obesity (Mariman et al., 2015). However, the precise function of BAIAP3 was unknown, so we characterized its cellular phenotype and its role in membrane trafficking.

Protein expression of BAIAP3 in BON cells was confirmed by Western blotting (Fig. 1 E). *BAIAP3* siRNAs reduced the expression of BAIAP3 without reducing the expression of CAPS (Fig. 1 E). We tested four different RNAi duplexes that targeted *BAIAP3* and found that three significantly inhibited NPY-Venus secretion (Fig. 1 F). In addition, endoribonuclease-prepared siRNAs (esiRNAs) targeting two regions of *BAIAP3* mRNA were found to generate a similar knockdown phenotype (Fig. S1 C). To eliminate off-target effects of siRNA knockdown, we conducted rescue studies. Overexpression of a siRNA-resistant *BAIAP3* partially rescued stimulated NPY-Venus secretion inhibited by siRNA (Fig. 1 G). Lack of full rescue was likely caused by incomplete transduction efficiency in expressing the siRNA-resistant *BAIAP3*. We used an orthogonal assay consisting of [^3^H]serotonin secretion stimulated by ionomycin and found that *BAIAP3* siRNA also inhibited Ca^2+^-induced [^3^H]serotonin secretion (Fig. 1 H). We noted that the reduced percent secretion of NPY-Venus or [^3^H]serotonin in BAIAP3 knockdown cells was mainly because of an increase in DCV cargo pool size (Fig. 1, I and J), which suggested that the number of DCVs may be increased by BAIAP3 knockdown (see the BAIAP3 knockdown caused accumulation of defective DCVs in BON cells section).

### BAIAP3 affects spontaneous DCV exocytosis

**Figure 2.**
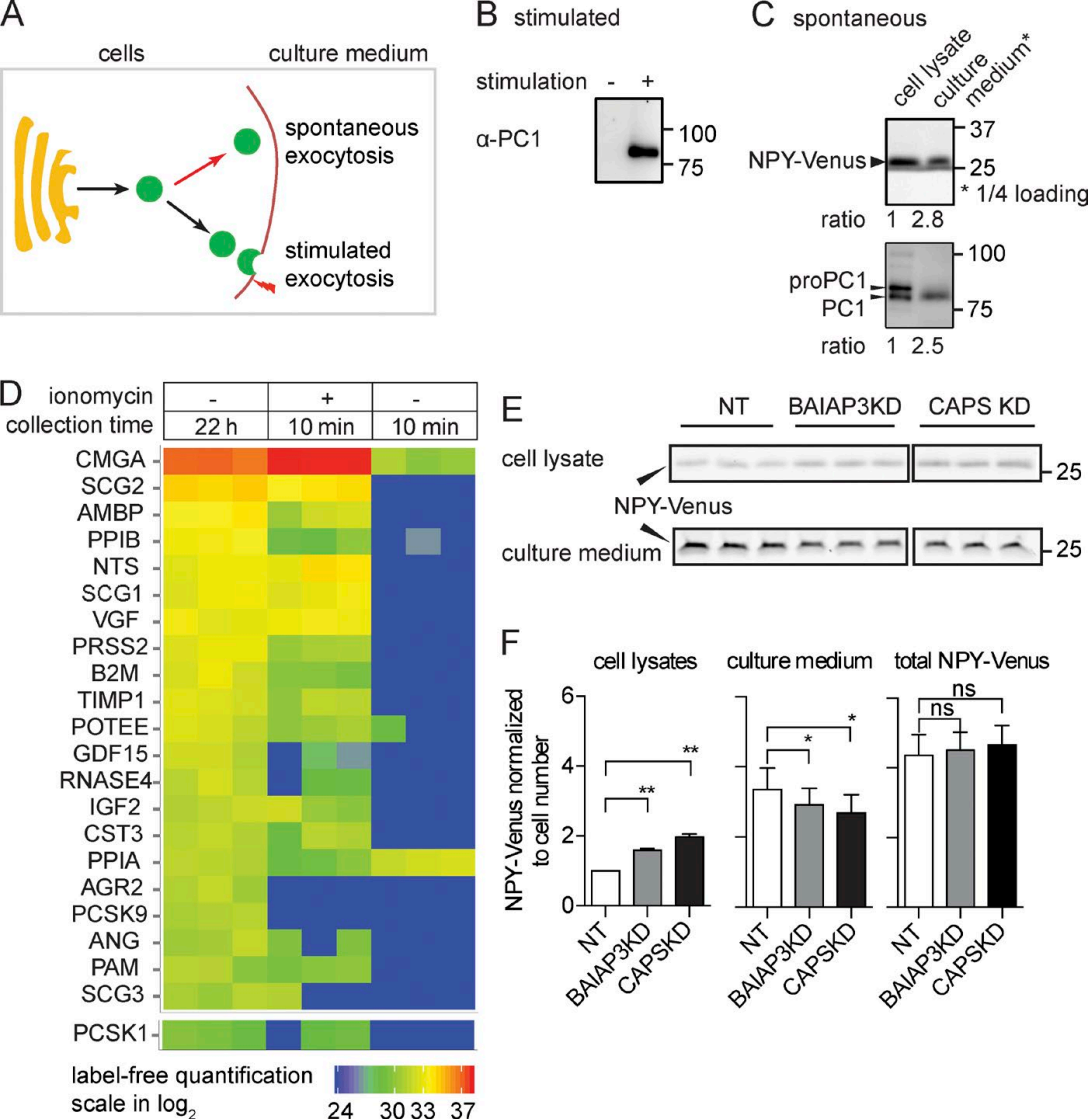
**BAIAP3 is required for spontaneous DCV exocytosis.** (A) Possible secretion pathways for NPY-Venus release: spontaneous DCV exocytosis at resting condition or stimulated exocytosis during stimulation. (B) Ca^2+^-triggered secretion of PC1. (C) NPY-Venus and PC1 in cell lysates and conditioned culture medium after 2 d. (D) Liquid chromatography tandem mass spectrometry analysis of spontaneously secreted proteins and acutely secreted proteins. Proteins are quantified by label-free quantification and ranked by their abundance in culture medium. *n* = 3 for each condition. (E) Spontaneous NPY-Venus secretion into culture medium from wild-type, BAIAP3 knockdown, or CAPS knockdown cells. *n* = 3. (B, C, and E) Molecular mass is shown in kilodaltons. (F) Quantification of NPY-Venus in cell lysates, culture medium, or total (culture medium + cell lysate) for conditions similar to E after normalizing to cell number. *n* = 3 independent experiments. See also Fig. S2. Data are expressed as mean ± SD. P-values were obtained by a two-tailed Student’s *t* test. *, P < 0.05; **, P < 0.01. KD, knockdown; NT, nontargeting.

An explanation for an increase of DCV cargo induced by BAIAP3 knockdown (Fig. 1 J) could be that spontaneous DCV exocytosis (Fig. 2 A) during the 2-d siRNA incubation was affected. Indeed, spontaneous DCV exocytosis was found to occur in BON cells based on the analysis of the 2 d–conditioned medium from control cells, which contained NPY-Venus as well as mature prohormone convertase 1 (PC1; PC1/PCSK1), which is a specific DCV cargo (Fig. 2, B and C; Carraway et al., 1994). Proteins secreted into the culture medium from control cells in 1 d were analyzed by liquid chromatography tandem mass spectrometry and compared with proteins secreted during an acute 10-min stimulation with ionomycin, which represents soluble DCV cargo proteins. Similar protein components (PC1/PCSK1, chromogranin A [CgA/CMGA] and derived peptides, secretogranin 1/2 [SCG1/2], neurotensin [NTS], and VGF nerve growth factor inducible [VGF]) were detected in both samples (Fig. 2 D and Fig. S2). Collectively, the results indicate that DCVs in resting BON cells undergo spontaneous exocytosis.

We assessed the effects of *BAIAP3* siRNA treatment over a 2-d interval on spontaneous DCV exocytosis by quantifying NPY-Venus in cell lysates and culture medium. *BAIAP3* siRNAs increased NPY-Venus in cell lysates and reduced amounts in the culture medium with minimal effects on total NPY-Venus (Fig. 2, E and F). The results indicate that the knockdown of BAIAP3 protein leads to the accumulation of DCV cargo in the cells.

### BAIAP3 knockdown caused accumulation of defective DCVs in BON cells

**Figure 3.**
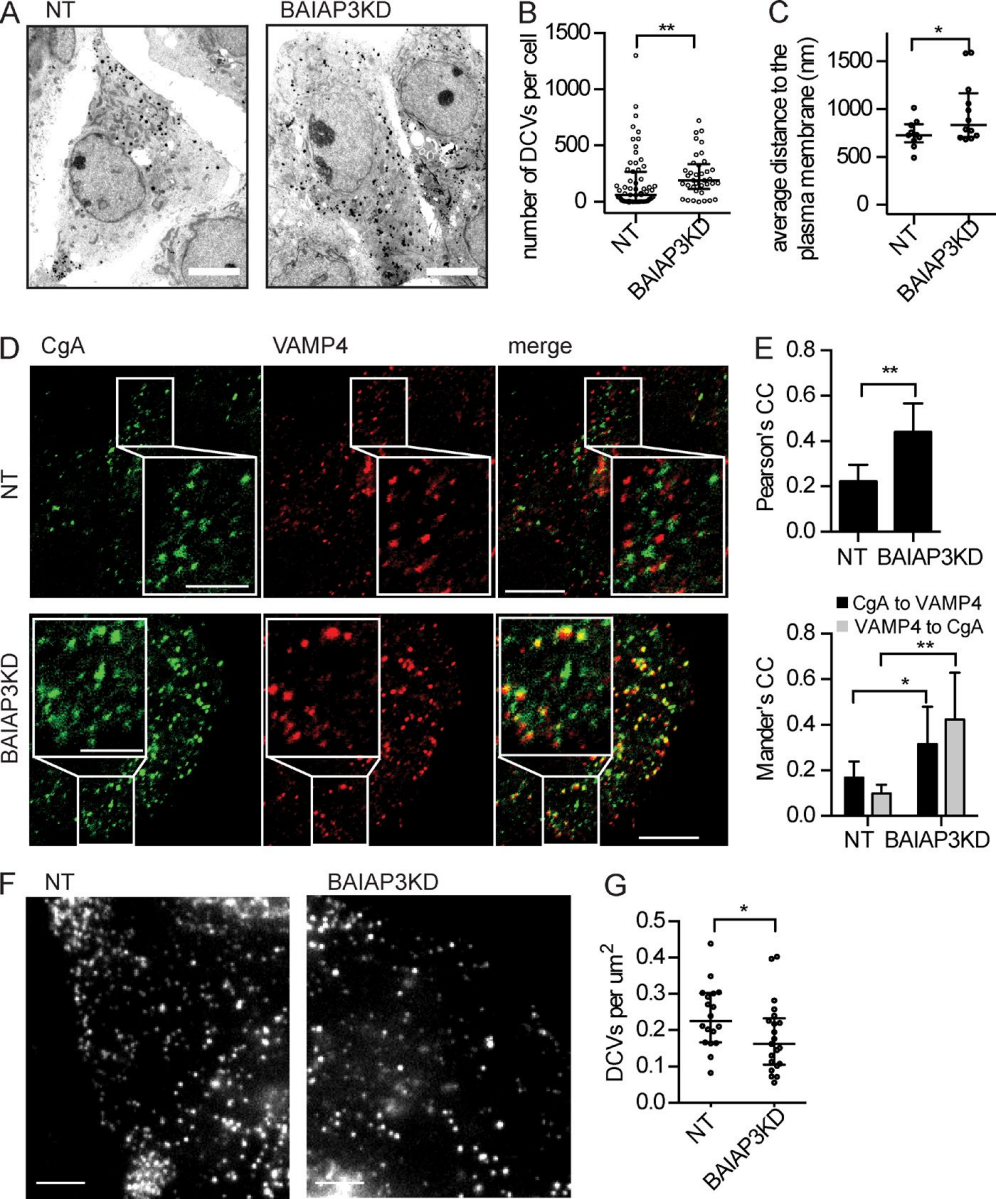
**Accumulation of defective DCVs in BON cells.** (A) Electron microscopy micrographs of BON cells. Bars, 5 µm. (B) Number of DCVs per electron microscopy section. *n* = 63 nontargeting (NT) and 37 knockdown (KD) cells. (C) Distance of DCVs to the cell border. *n* = 10 nontargeting and 12 knockdown cells. (D) Immunofluorescent staining of CgA and VAMP4. Bars: 10 µm; (inset) 5 µm. (E) Colocalization analysis of CgA and VAMP4. *n* = 8. CC, correlation coefficient. (F) NPY-Venus^+^ DCVs in the TIRF field. Bar, 5 µm. (G) Number of DCVs per square micrometer in the TIRF field. *n* = 18 nontargeting and 21 knockdown. See also Fig. S3. (B, C, and G) Data are expressed as median ± quantile. P-values were obtained by Wilcoxon test. (E) Data are expressed as mean ± SD. P-values were obtained by a two-tailed Student’s *t* test. *, P < 0.05; **, P < 0.01.

The intracellular accumulation of DCV cargo during the *BAIAP3* siRNA incubation might be because of an increased number of DCVs. Electron microscopy of BON cells confirmed that there were 39% more DCVs [mean value] after BAIAP3 knockdown (Fig. 3, A and B). However, despite the increased pool of DCVs, acute ionomycin stimulation of BAIAP3 knockdown cells resulted in amounts of secretion comparable with that of control cells (Fig. 1 I), which indicated that DCVs accumulated during the 2-d BAIAP3 knockdown were defective for exocytosis. Because immature DCVs respond poorly to stimuli (Tooze et al., 1991; Eaton et al., 2000; Kögel and Gerdes, 2010; Bonnemaison et al., 2013), we determined whether BAIAP3 knockdown cells had an increased number of immature DCVs. Golgi VAMP4 is present on immature but not mature DCVs (Eaton et al., 2000). Only a small fraction of CgA^+^ DCVs colocalized with VAMP4 in control cells, but this significantly increased after BAIAP3 knockdown (Fig. 3, D and E), indicating an increased number of immature DCVs. In addition, a small reduction in the mean size of DCVs was detected by electron microscopy (Fig. S3 A), consistent with a DCV maturation defect (Tooze et al., 1991). A defect in DCV maturation likely explains the accumulation of DCVs during the 2-d *BAIAP3* siRNA treatment.

In addition to a maturation defect, DCV trafficking was disrupted in BAIAP3 knockdown cells. In electron micrographs of control cells, most DCVs were close to the plasma membrane and enriched in cell protrusions (Fig. 3 A). In contrast, DCVs in BAIAP3 knockdown cells were more randomly distributed throughout the cytoplasm (Fig. 3, A and C). To specifically view plasma membrane–proximal DCVs, we imaged cells by total internal reflection fluorescence (TIRF) microscopy and found plasma membrane–proximal DCVs to be significantly reduced in BAIAP3 knockdown cells compared with control cells (Fig. 3, F and G), even though the total number of DCVs in the cells was increased (Fig. 3 B). The results indicate that BAIAP3 knockdown in resting cells caused DCV trafficking defects, which could indicate that the accumulated defective DCVs lack key proteins required for DCV transport on the cytoskeleton.

### BAIAP3 knockdown caused a loss of insulin granules

**Figure 4.**
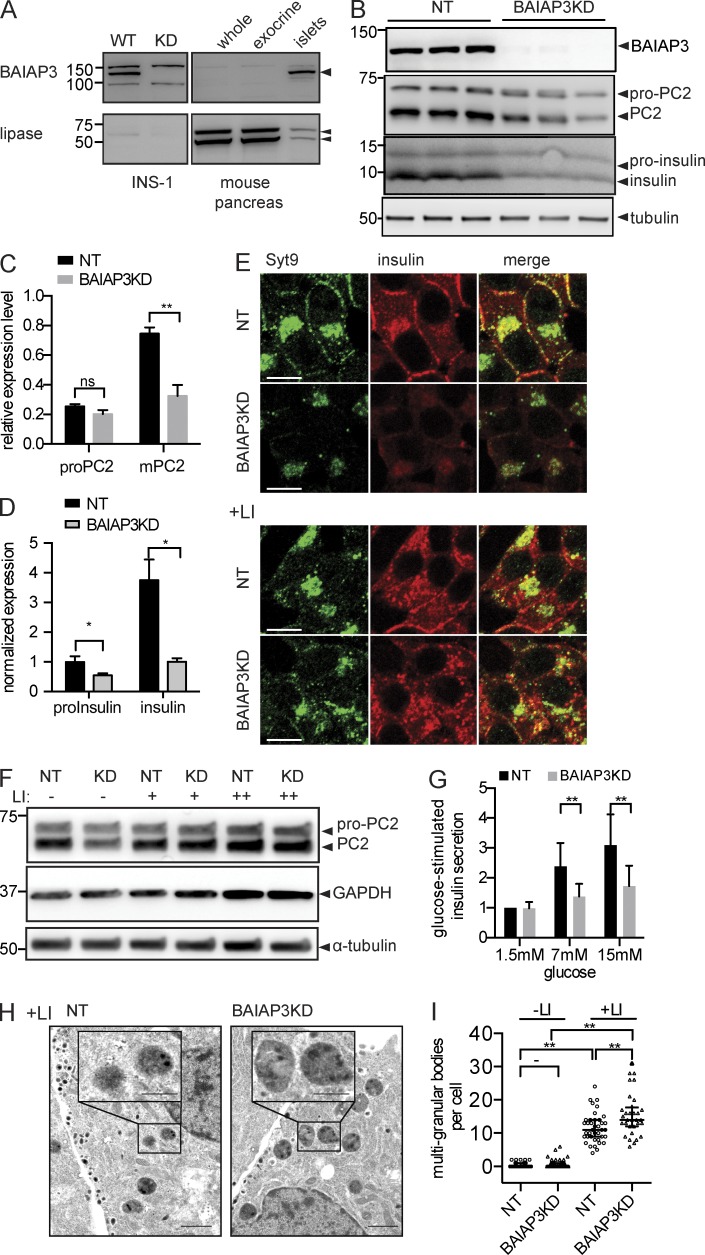
**Loss of insulin granules in INS-1 cells.** (A) Endogenous BAIAP3 in INS-1 cells and pancreas. (B) Loss of PC2 and insulin after BAIAP3 knockdown (BAIAP3KD) in INS-1 cells. *n* = 3. KD, knockdown; NT, nontargeting. (C and D) Quantification of PC2 (C) and insulin (D) normalized to tubulin. *n* = 3. (E) Loss of insulin granules after BAIAP3 knockdown and rescue with lysosome inhibitors (LI; pepstatin A, 10 µg/ml; E64D, 10 µg/ml). Bars, 10 µm. (F) Rescue of PC2 with lysosome inhibitors. + = 1 µg/ml; ++ = 10 µg/ml. (A, B, and F) Molecular mass is shown in kilodaltons. (G) Glucose-induced insulin secretion. Basal release (1.5 mM glucose) from nontargeting cells is set as 1. *n* = 6. (H) Electron microscope analysis of INS-1 cells in the presence of lysosome inhibitors. Bars:1 µm; (inset) 0.5 µm. (I) Counting of multigranular bodies in INS-1 cells. *n* = 35, 72, 39, and 32 cells from left to right. See also Fig. S3. (C, D, and G) Data are expressed as mean ± SD. P-values were obtained by a two-tailed Student’s *t* test. (I) Data are expressed as median ± quantile. P-values were obtained by Wilcoxon test. *, P < 0.05; **, P < 0.01.

To determine whether BAIAP3 knockdown affected DCV maturation in other cell types, we conducted studies in pancreatic β cell lines. BAIAP3 is expressed at relatively high levels in the β cell line INS-1 832/13 (called INS-1) and in primary pancreatic islets but not in pancreatic exocrine cells (Fig. 4 A). Efficient BAIAP3 knockdown in INS-1 cells dramatically reduced the cellular content of PC2 (PC2/PCSK2) and insulin (Fig. 4, B–D). However, proinsulin and pro-PC2, which reside in the Golgi, were reduced to a much lesser extent or not at all. The preferential reduction of mature PC2 and insulin suggested a loss of insulin granules, which was confirmed by finding a strong reduction of Syt-9, an insulin granule membrane marker (Fig. 4 E, top; Iezzi et al., 2004), and also by electron microscope imaging (Fig. S3 B). Residual insulin granules in BAIAP3 knockdown cells were also significantly smaller (by 18%) in electron microscopy (Fig. S3 C). Consistent with the loss of insulin granules, glucose-stimulated insulin secretion was markedly reduced in BAIAP3 knockdown cells (Fig. 4 G). The loss of insulin granules could be caused by enhanced spontaneous DCV exocytosis, by reduced DCV biogenesis, or by increased DCV turnover. We did not detect a significant increase of insulin or proinsulin in cell culture medium from BAIAP3 knockdown cells (not depicted), which excludes increased spontaneous release as responsible. However, blocking degradation with lysosomal protease inhibitors resulted in a restoration of PC2/PCSK2 levels (Fig. 4 F) as well as of Syt-9– and insulin-immunoreactive structures (Fig. 4 E, bottom). Electron microscopy (Fig. 4, H and I) revealed that these structures were multigranular bodies that are characteristic of crinophagy or microautophagy (Orci et al., 1984; Marsh et al., 2007). The results indicate that INS-1 cells with BAIAP3 knockdown generate defective insulin granules that are preferentially targeted for lysosomal degradation.

### BAIAP3 localizes to late and recycling endosomes

**Figure 5.**
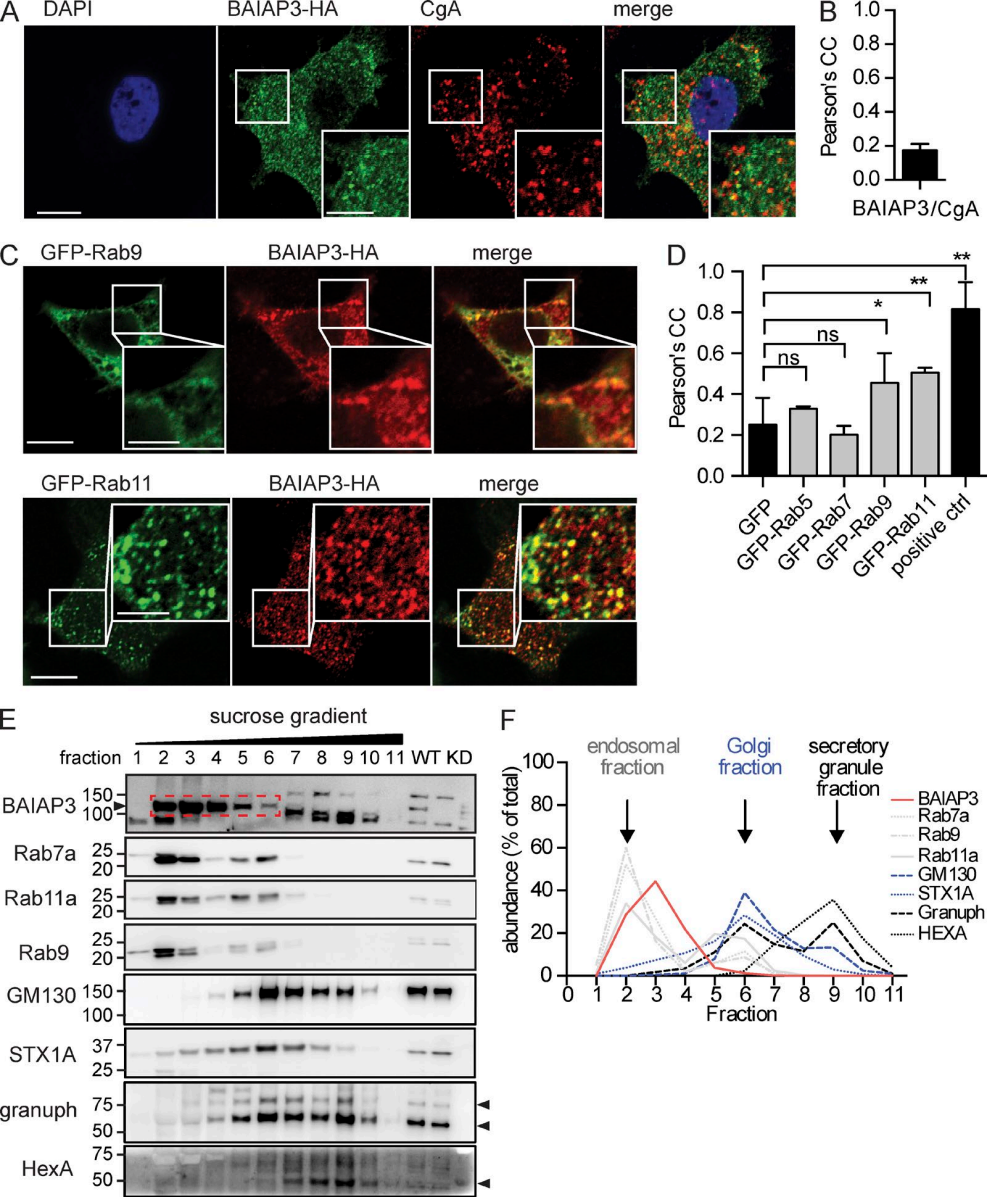
**BAIAP3 localization to endosomes.** (A) Coimmunofluorescent staining of BAIAP3-HA and DCV marker CgA. (B) Colocalization analysis of BAIAP3-HA and CgA. *n* = 6 regions. (C) Immunofluorescent staining of BAIAP3-HA in cells expressing GFP-Rab9 or -Rab11. (A and C) Bars: 10 µm; (inset) 5 µm. (D) Colocalization analysis of BAIAP3-HA and Rabs. GFP is used as the negative control, and the self-colocalization of Rab7 (GFP and mCherry tagged) is the positive control (ctrl). *n* = 9, 3, 7, 7, 4, and 8 regions. (E) INS-1 cell fractionation and equilibrium centrifugation on sucrose gradient (0.27–2 M). WT and BAIAP3 knockdown cell lysates were loaded as the control in the last two lanes. granuph, granuphilin. Arrowheads indicate expected bands for the corresponding blot. BAIAP3 bands are also circled in the red dashed box. Molecular mass is shown in kilodaltons. (F) Distribution of organelle markers in the sucrose gradient. Each line corresponds to the respective blot shown in E. See also Fig. S4. Data are expressed as mean ± SD. P-values were obtained by a two-tailed Student’s *t* test. *, P < 0.05; **, P < 0.01.

To determine how BAIAP3 knockdown caused the generation of defective DCVs, we determined the cellular localization of BAIAP3. C-terminal HA-tagged BAIAP3 mainly localized to cytoplasmic punctate structures in BON cells that did not colocalize with the DCV marker CgA (Fig. 5, A and B). However, we found substantial colocalization of BAIAP3-HA with GFP-Rab11^+^ recycling endosomes and with GFP-Rab9^+^ late endosomes. In contrast, BAIAP3-HA did not colocalize with GFP-Rab5^+^ early endosomes or GFP-Rab7^+^ late endosomes (Fig. 5, C and D). Rab9 and Rab7 occupy different subdomains of the late endosome (Barbero et al., 2002), which could account for differences in the colocalization of BAIAP3 with late endosomal Rab proteins.

To confirm an endosomal location for BAIAP3, we fractionated INS-1 cells and separated cellular organelles on equilibrium sucrose gradients. BAIAP3 migrated to light fractions, which were positive for the endosomal markers Rab7, Rab11, and Rab9, but not to heavy fractions, which were positive for the DCV marker α-granuphilin (Fig. 5, E and F). Endogenous BAIAP3 also localized to intracellular punctate structures in INS-1 cells (Fig. S4), similar to BAIAP3-HA overexpressed in BON cells. We conclude that BAIAP3 localizes to recycling and late endosomes but not to DCVs.

### BAIAP3 promotes endosome recycling to the TGN

**Figure 6.**
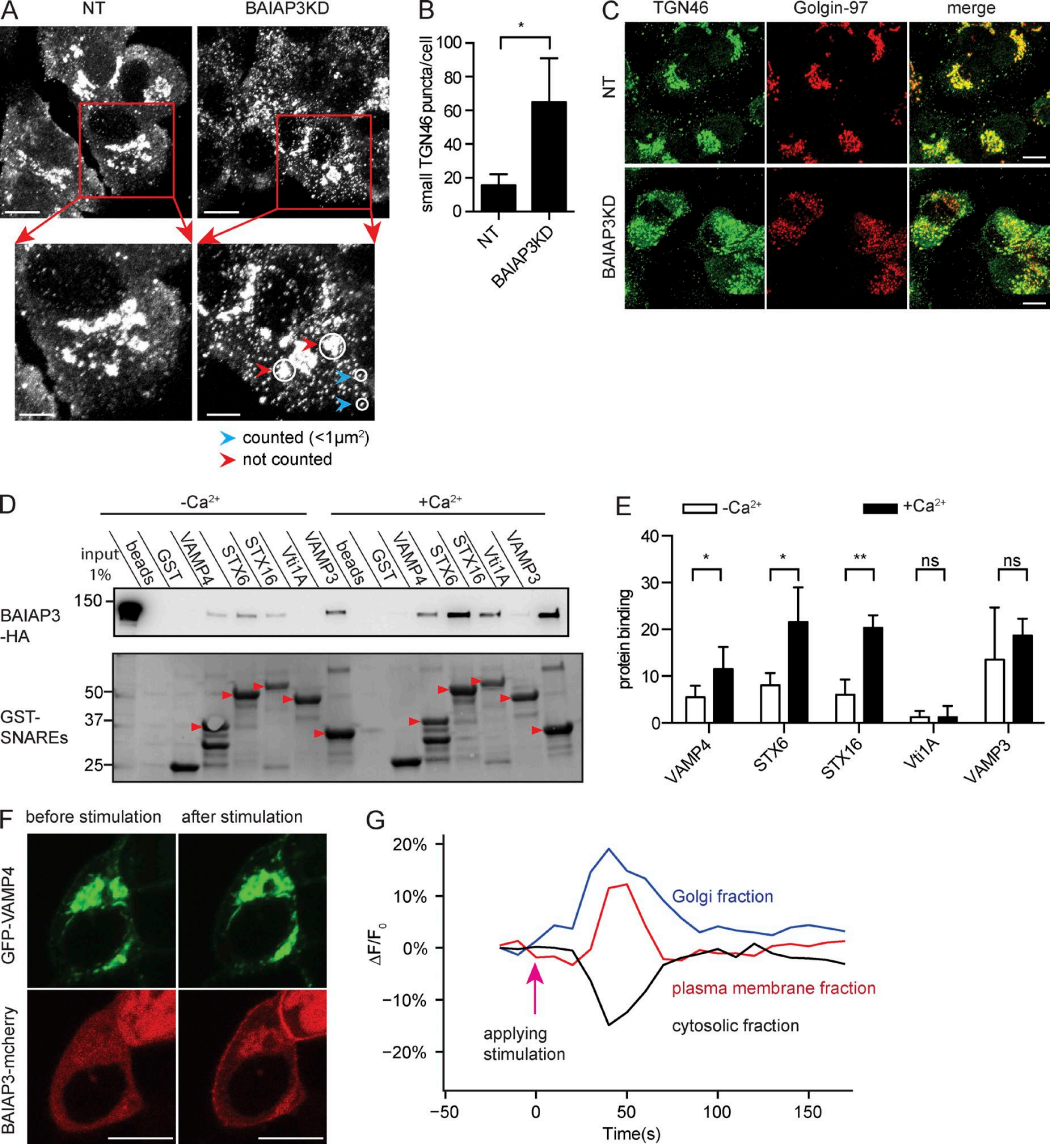
**BAIAP3 functions in retrograde trafficking.** (A) Representative images of TGN46 in BON cells. Bars: 10 µm; (inset) 5 µm. BAIAP3KD, BAIAP3 knockdown; NT, nontargeting. (B) Quantification of small puncta of TGN46. *n* = 4. (C) Coimmunofluorescent staining of TGN46 and Golgin-97. Bar, 10 µm. (D) GST pulldown of BAIAP3-HA from BON cell lysates by immobilized cytosolic domains of SNAREs in the presence of 100 µM Ca^2+^ or 1 mM EGTA/EDTA. Arrowheads indicate the expected protein bands. Molecular mass is shown in kilodaltons. (E) Ca^2+^-enhanced pulldown by different SNAREs. Protein binding is determined by normalizing the density of each BAIAP3-HA band to total BAIAP3-HA pulled down by all SNAREs within one experiment. *n* = 6. (F) Ionomycin stimulation induced BAIAP3 recruitment to the TGN and the plasma membrane in BON cells coexpressing BAIAP3-mCherry and GFP-VAMP4. See also Video 1. Bars, 10 µm. (G) Quantification of BAIAP3 partition in response to ionomycin stimulation. See also Figs. S5 and S6. Data are expressed as mean ± SD. P-values were obtained by a two-tailed Student’s *t* test. *, P < 0.05; **, P < 0.01.

The preceding results show that defective DCVs are generated in cells lacking BAIAP3. This could indicate that endosome-localized BAIAP3 functions in a recycling process that retrieves DCV proteins (e.g., VAMP4) from immature DCVs or that recycles DCV proteins that are lost to the plasma membrane during exocytosis (Houy et al., 2013). To test whether BAIAP3 functions in retrograde trafficking, we assessed the localization of TGN46, which recycles to the TGN via early and recycling endosomes (Johannes and Popoff, 2008). TGN46 mainly localized to the perinuclear TGN in control cells but was widely distributed in punctate structures in BAIAP3 knockdown cells (Fig. 6, A and B), consistent with a lack of recycling back to the TGN. Perinuclear TGN46 in control cells and cytoplasmic TGN46 in BAIAP3 knockdown cells colocalized with the TGN marker Golgin-97 (Fig. 6 C), indicating that TGN integrity was disrupted after BAIAP3 knockdown. Disruption of steady-state TGN46 localization by BAIAP3 knockdown was quite similar to that observed for the depletion of other proteins required for retrograde trafficking to the TGN, such as VAMP4, STX6, STX16, Vti1A, conserved oligomeric Golgi 6 (COG6) of the COG complex, and VPS52 of the Golgi-associated retrograde protein (GARP) complex (Ganley et al., 2008; Pérez-Victoria et al., 2008; Laufman et al., 2011; Shitara et al., 2013).

We addressed the possible mechanism by which BAIAP3 could promote endosome recycling to the TGN. Other Munc13 proteins exhibit phospholipid- and SNARE-binding properties that mediate membrane fusion reactions (Guan et al., 2008; Boswell et al., 2012; James and Martin, 2013; Woo et al., 2017). As BAIAP3 localizes to Rab11^+^ and Rab9^+^ endosomes, which can fuse with the TGN, we anticipated that BAIAP3 could interact with SNAREs and lipids on the TGN. The fusion of recycling endosomes with the TGN utilizes SNARE complexes formed by STX6, STX16, Vti1A, and VAMP3 or VAMP4 (Mallard et al., 2002). GST-tagged cytoplasmic domains of STX6, STX16, VAMP3, and VAMP4 but not Vti1A were found to retain BAIAP3-HA from cell lysates (Fig. 6, D and E). BAIAP3 contains two C2 domains predicted to bind Ca^2+^ (Fig. S6), and we found that Ca^2+^ increased STX6, STX16, and VAMP4 interactions with BAIAP3 (Fig. 6, D and E). In a protein-lipid overlay assay (Fig. S5), recombinant BAIAP3 bound to PI(4)P and PI(3)P, characteristic of Golgi and endosomal membranes, respectively. Collectively, the results suggest that BAIAP3 could promote SNARE complex assembly on PI(4)P-containing membranes to mediate retrograde trafficking to the TGN.

Lastly, we assessed the Ca^2+^-dependent properties of BAIAP3 in live cells. mCherry-BAIAP3 was mainly cytoplasmic with some punctate structures in resting BON cells. When intracellular Ca^2+^ levels were increased by ionomycin stimulation, BAIAP3 was rapidly recruited to the plasma membrane and to GFP-VAMP4^+^ Golgi structures but not to DCVs (Fig. 6, F and G and Video 1). After ∼60 s, mCherry-BAIAP3 at the Golgi returned to the resting level before stimulation. The recruitment of BAIAP3 to the Golgi is consistent with a proposed role in promoting endosome fusion with the TGN in retrograde trafficking. Recruitment to the plasma membrane was consistent with previous studies (Lecat et al., 2015). Whether BAIAP3 also functions in endocytic retrieval of exocytosed DCVs at the plasma membrane needs further investigation.

## Discussion

### C2-domain proteins in the regulated secretory pathway

Several C2 domain–containing proteins function in Ca^2+^-dependent vesicle exocytosis (Südhof, 2012; Pinheiro et al., 2016). We report the first systematic screen of C2 domain–containing proteins in the regulated secretory pathway of neuroendocrine cells. Ionomycin directly mediates Ca^2+^ entry so that the screen only identified proteins that exert their actions downstream of Ca^2+^ entry. The quality of the screen was verified by multiple controls and by the successful identification of genes for proteins known to function in regulated exocytosis as the top hits *CADPS* and *UNC13B* (Table S2). Other strong hits involved in regulated exocytosis were *RIMS1* encoding a Rab3- and Munc13-1–interacting protein (Deng et al., 2011), *SYTL1/2* encoding Rab effectors involved in DCV docking (Fukuda, 2013), *DOC2A* encoding a protein required for DCV exocytosis (Li et al., 2014), and *SYT10* encoding a Ca^2+^ sensor for peptidergic granule fusion (Cao et al., 2011). These proteins operate directly in the docking, priming, and fusion steps of DCV exocytosis. In contrast, the knockdown of other C2-domain proteins appeared to affect DCV exocytosis indirectly through other trafficking pathways. *PLA2* siRNAs strongly reduced the percent secretion in the screen, possibly by affecting cargo protein trafficking to the plasma membrane by altering Golgi tubulation (Ha et al., 2012). A large number of hits likely impacted NPY-Venus secretion by affecting endocytosis (*ITSN1*, *TOLLIP*, and *MCTP1/2*) or endosomal trafficking (*RAB11FIP2/5*, *MYOF*, *FER1L5*, *FER1L6*, and *DYSF*; Shin et al., 2005; Tarbutton et al., 2005; Capelluto, 2012; Gubar et al., 2013; Qiu et al., 2015; Redpath et al., 2016). The latter group may function at intersections between the regulated secretory pathway and endosomal trafficking by affecting DCV biogenesis, maturation, or recycling. Lastly, siRNAs directed against several targets (*CPNE4/7*, *PLCB1*, *PLCL1*, *PRKCA*, *CAPN6*, and *ESYT2/3*) likely involve Ca^2+^-dependent signaling that affects posttranslational modifications of proteins and cytoskeletal elements in the regulated secretory pathway (Tonami et al., 2011; Sugiyama et al., 2013; Reinhard et al., 2016; Yu et al., 2016). Overall, the screen provided a foundation for characterizing novel C2 domain–containing proteins for their direct or indirect roles in the regulated secretory pathway.

### BAIAP3 controls the fate of DCVs

The screen identified *BAIAP3* as a novel gene required for optimal function in the regulated secretory pathway. BAIAP3 is a paralog of Munc13-4, but the tissue expression of these proteins differs, with BAIAP3 expressed predominantly in the hypothalamus and other brain regions, the pituitary, pancreatic islets (this study), and in natural killer and CD8^+^ T cells, whereas Munc13-4 is predominantly expressed in secretory myeloid cells (Genevestigator, GTExPortal: *BAIAP3* or *UNC13D*). Mouse and human genetic studies suggested a role for BAIAP3 in the nervous system in regulating behavior and food intake (Lauridsen et al., 2011; Wojcik et al., 2013; Mariman et al., 2015). BAIAP3 was also proposed to participate in growth factor secretion by tumor cells (Palmer et al., 2002). Our results provide evidence that BAIAP3 indirectly affects DCV-mediated peptide secretion.

**Figure 7.**
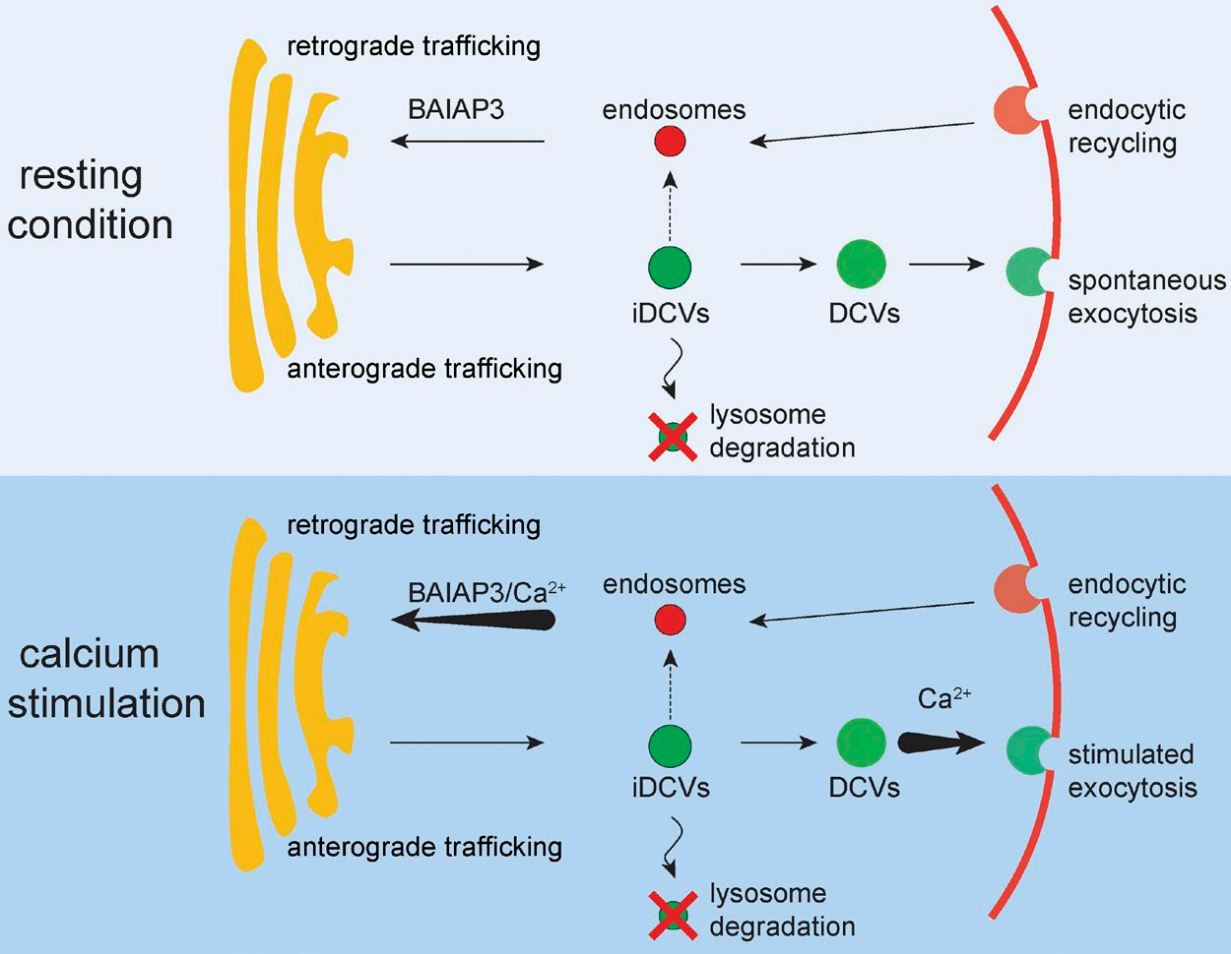
**Proposed model for BAIAP3 function.** (Top) In resting cells, DCVs undergo maturation and slow spontaneous exocytosis. DCV membrane proteins are recycled to the TGN by retrograde trafficking with the basal activity of BAIAP3. Knockdown of BAIAP3 disrupts protein recycling and may deplete DCV membrane proteins, causing DCV maturation and trafficking defects that lead to immature DCV (iDCVs) accumulation in BON cells or DCV turnover in INS-1 cells. (Bottom) When cells are stimulated with Ca^2+^, increased BAIAP3 activity may stimulate retrograde trafficking to meet the increased demand for protein recycling during Ca^2+^-triggered DCV exocytosis.

The detection of endogenous DCV cargo, especially mature peptide hormones that are only processed within DCVs, in conditioned medium indicated that basal secretion in BON cells was mediated by spontaneous DCV exocytosis (Fig. 2 D and Fig. S2), as reported for other peptides in endocrine cells (Mains and Eipper, 1984; Sambanis et al., 1990; Matsuuchi and Kelly, 1991; Sirkis et al., 2013). *BAIAP3* siRNA appeared to block vesicle maturation, which was reflected by increased VAMP4 retention on DCVs, decreased DCV size, and disrupted DCV trafficking. Immature DCVs accumulated in resting cells over 2-d incubations with BAIAP3 siRNAs as a result of spontaneous DCV exocytosis with suboptimal recycling (Fig. 7, top). When challenged with Ca^2+^/ionomycin, the accumulated DCVs failed to release cargo, resulting in decreased percent secretion. A parsimonious explanation of the results in BON cells is that DCVs accumulated during the 2 d of *BAIAP3* siRNA treatment were defective for exocytosis, whereas other DCVs participated in exocytosis, thereby decreasing percent secretion. Thus, BAIAP3 knockdown did not affect exocytosis per se but conferred a loss of function to a pool of DCVs produced during the siRNA incubation. Our results are compatible with a study in chromaffin cells from BAIAP3 knockout mice, indicating that the Ca^2+^-triggered exocytosis of a small rapid release pool of DCVs was not affected (Man et al., 2015), but the status of other DCVs in the chromaffin cells was not assessed.

The fate of DCVs was different in INS-1 cells compared with BON cells after BAIAP3 knockdown (Fig. 7). The greater efficiency of BAIAP3 knockdown in INS-1 cells may be part of the reason. Nonetheless, BAIAP3 knockdown in INS-1 cells caused a dramatic decrease in insulin granules by promoting their lysosomal degradation (Fig. 4). Insulin granules undergo spontaneous exocytosis both in primary β cells (Del Prato et al., 2002) and in INS-1 cells (Fig. 4 G, 1.5 mM glucose). Therefore, BAIAP3 knockdown for 2 d may similarly disrupt the recycling of insulin granule membrane proteins after spontaneous exocytosis. Insulin granules did not accumulate during the 2-d BAIAP3 knockdown, likely because of an efficient stress response in β cells that targets defective insulin granules for crinophagy or microautophagy (Orci et al., 1984; Marsh et al., 2007; Goginashvili et al., 2015). For example, insulin granules lacking Rab3, a protein that recycles from DCVs through an endosomal intermediate to the TGN (Jena et al., 1994; Slembrouck et al., 1999), undergo lysosomal degradation (Marsh et al., 2007). Although the fate of DCVs is different in BON and INS-1 cells, the mechanisms underlying the generation of defective DCVs in cells lacking BAIAP3 are likely similar, involving the Golgi biogenesis of DCVs with an altered balance of membrane proteins such that mature functional DCVs are not generated.

### BAIAP3 plays a role in retrograde trafficking

Lack of localization to DCVs implied that BAIAP3 might not play a direct role in DCV exocytosis. Our evidence indicated that endosome-localized BAIAP3 played a role in endosome-mediated retrograde trafficking. BAIAP3 knockdown strongly disrupted the steady-state localization of TGN46 as well as TGN integrity, which is similar to the knockdown phenotype of proteins required for retrograde trafficking such as COG and GARP complexes (Pérez-Victoria et al., 2008; Laufman et al., 2011) or the SNARE proteins VAMP4, STX6, STX16, and Vti1A (Ganley et al., 2008; Shitara et al., 2013). Retrograde trafficking plays an important role in the DCV cycle. When DCVs undergo exocytosis, transmembrane DCV proteins such as phogrin (Vo et al., 2004), peptidylglycine α-amidating monooxygenase (Bäck et al., 2010), carboxypeptidase E (Arnaoutova et al., 2003), VAMP2, and synaptotagmin (Cárdenas and Marengo, 2016) are lost to the plasma membrane and need to be recycled to support new DCV biogenesis (Bauer et al., 2004). Proteins such as STX6, VAMP4, and SYT4 are transient residents on immature DCVs that are recycled to the TGN during DCV maturation (Bonnemaison et al., 2013). The recycling of VAMP4 from DCVs in BON cells was deficient in BAIAP3 knockdown cells. Collectively, the results indicate that BAIAP3 plays an important role in endosome-mediated protein recycling, which indirectly affects the regulated secretory pathway.

### Coupling of retro- and anterograde trafficking

Because of the coupling of retrograde and anterograde trafficking, the disruption of retrograde trafficking by BAIAP3 knockdown could deplete essential proteins in the TGN, leading to the generation of defective DCVs that fail to mature or are subjected to degradation by crinophagy or microautophagy. There are several examples where the perturbation of endosomal proteins exert effects on DCV function, such as for the knockdown of endosomal AP3 (Asensio et al., 2010) or the knockout of Vti1A (Walter et al., 2014), which impair DCV biogenesis and maturation. Knockdown of the endosomal proteins VAMP3, Rab11FIP2/5, and ITSN2 also disrupted NPY-Venus secretion in our screen. These studies reveal the significance of endosome-mediated retrograde trafficking for DCV-mediated anterograde trafficking.

BAIAP3 contains a CATCHR (complex associated with tethering containing helical rods) domain found in GARP and COG tethering complexes, which are required for endosome fusion with the TGN (Pei et al., 2009; James and Martin, 2013; Hong and Lev, 2014). Similarly to COG and GARP (Hong and Lev, 2014), BAIAP3 interacts with SNARE proteins (STX6, STX16, VAMP3, and VAMP4) required for endosome-mediated retrograde trafficking. Thus, BAIAP3 may function in resting cells in parallel with COG and GARP complexes to enable endosome-TGN trafficking especially for DCVs retrieved after spontaneous exocytosis. As a paralog of Munc13-4, BAIAP3 likely also exhibits Ca^2+^-stimulated activity at endosome-TGN interfaces. Indeed, we found that BAIAP3 interactions with TGN SNAREs was enhanced by Ca^2+^ and that BAIAP3 was recruited to the TGN in response to Ca^2+^ elevations. Because BAIAP3 is expressed in cells with a regulated secretory pathway, Ca^2+^ increases could activate the C2 domains of BAIAP3 to enhance retrograde trafficking in concert with the Ca^2+^-dependent activation of the C2 domains of Munc13-2 and SYT10, which accelerate DCV exocytic trafficking. Thus, BAIAP3 may serve as a key Ca^2+^-dependent effector in retrograde trafficking that functions to meet the increased demand for protein recycling during Ca^2+^-stimulated DCV exocytosis in secretory cells (Fig. 7, bottom).

An alternative possibility is that BAIAP3 controls protein exchange between endosomes and immature DCVs without the need to recycle proteins to the TGN. In this model, transient proteins retrieved from immature DCVs are delivered to endosomes, and DCV membrane proteins recycled from the plasma membrane are delivered to immature DCVs by endosomes. This model was partly supported by studies of Rab2 and its effectors in *Caenorhabditis elegans* (Edwards et al., 2009; Hannemann et al., 2012; Ailion et al., 2014) and could explain some of our results. The knockdown of BAIAP3 may cause loss of proteins from immature DCVs or a failure to deliver essential proteins for exocytosis. A careful examination of possible two-way traffic between endosomes and DCVs is needed to test this model.

In conclusion, we identified BAIAP3 as essential for optimal function in the regulated secretory pathway (Fig. 7). However, BAIAP3 acts on endosomal pathways to control the fate and activity of DCVs in endocrine and neuroendocrine cells. We propose that BAIAP3 functions in retrograde trafficking to maintain a homeostatic balance of a critical subset of DCV transmembrane proteins during their recycling. BAIAP3 may function similarly to other Munc13 proteins in regulating SNARE-dependent fusion but, unlike other Munc13 proteins, appears to uniquely regulate membrane fusion at an endosome-TGN interface.

## Materials and methods

### Cell culture

BON cells were a gift from C.M. Townsend (University of Texas Medical Branch, Galveston, TX). BON cells were maintained in DMEM/F12 (1:1; cat nos. 11965-092 and 11765-062; Life Technologies) supplemented with 10% FBS (cat no. FBS-500US; Phenix Research) at 37°C with 5% CO_2_. HEK293FT cells were maintained in DMEM supplemented with 10% FBS (cat no. 16000044; Life Technologies) at 37°C with 5% CO_2_. INS-1 832/13 cells were maintained in RPMI-1640 medium (cat no. 31800-022; Life Technologies) supplemented with an additional 2 mM l-glutamine, 1 mM Na-pyruvate, 0.000352% β-mercaptoethanol, and 10% FBS (cat. no. 16000044; Life Technologies) at 37°C with 5% CO_2_.

### Reagents

#### Antibodies

The following antibodies were used for Western blotting. CAPS/CADPS antibody was raised against the whole protein in rabbit, purified by protein A–agarose chromatography, and used at a 1:1,000 dilution. PC1 and PC2 antibodies were gifts from N. Seidah (University of Montreal, Quebec, Canada) and used at 1:5,000 and 1:20,000 dilutions. Munc13-4 antibody was a gift from H. Horiuchi (Tohoku University, Sendai, Japan) and used at 1:1,000 dilution. Tubulin antibody was purchased from Proteintech (cat. no. 10068-1-AP) and used at 1:2,000 dilution. GAPDH and Rab9 antibodies were purchased from Life Technologies (cat. nos. AM4300 and Ma3-067) and used at 1:4,000 and 1:2,000 dilutions. STX1A HPC1 antibody was purchased from Sigma (cat. no. WH0006804M1-100UG) and used at 1:2,500 dilution. VAMP2 and BAIAP3 antibodies were purchased from Synaptic Systems (cat. nos. 104202 and 256003) and used at 1:1,000 dilution. Insulin antibody was purchased from DAKO (cat. no. A056401-2) and used at 1:2,000 dilution. Rab7a was purchased from Abcam (cat. no. 50533) and used at 1:2,000 dilution. Rab11a and GM130 were purchased from BD Biosciences (cat. nos. 610656 and 610822) and used at 1:1,000 and 1:500 dilutions. Granuphilin and HexA antibodies were purchased from Santa Cruz Biotechnology (cat. nos. sc-34446 and sc-48530) and used at 1:200 dilution.

The following antibodies were used for immunofluorescent staining. HA and Golgin-97 antibodies were purchased from Cell Signaling Technology (cat. nos. 3724P and 13192S) and used at 1:1,600 and 1:100 dilutions. VAMP4 antibody was purchased from Synaptic Systems (cat. no. 136002) and used at 1:100 dilution. TGN46 antibody was purchased from AbD Serotec (cat. no. AHP500GT) and used at 1:500 dilution. BAIAP3 antibody was purchased from Synaptic Systems (cat. no. 256003) and used at 1:500 dilution. CgA antibody was purchased from Santa Cruz Biotechnology (cat. no. sc-1488) and used at 1:50 dilution. Insulin antibody was purchased from DAKO (cat. no. A056401-2) and used at 1:200 dilution. Syt IX antibody was purchased from BD Biosciences (cat. no. 612284) and used at 1:100 dilution.

#### Plasmids

NPY-Venus construct encoding human prepro-NPY (without C peptide) tagged with Venus was inserted into the BamHI/EcoRI sites of lentiviral vector pWPXL (cat. no. 12257; Addgene). TfR-mCherry lentiviral vector was purchased from Clontech (cat. no. 632580). BAIAP3 was cloned from its cDNA (cat. no. MHS4426-99240349; Dharmacon) and inserted into a lentiviral vector. An HA epitope sequence was added to the C terminus of BAIAP3 before the stop codon. mCherry-BAIAP3 construct was generated by inserting the mCherry in an N-terminal structurally flexible region after E108, and the construct was made with a pEGFP vector. GFP-Rab5 and GFP-Rab11 were generated by inserting canine Rab5 and Rab11 coding sequences into KpnI and BamHI sites of pEGFP-C1. GFP-Rab7 and GFP-Rab9 plasmids were purchased from Addgene (cat. nos. 12605 and 12663) and modified to remove the HA-coding sequence within two KpnI sites. The shRNAs were generated by inserting the seed sequence into pLKO.1 (cat. no. 10878; Addgene) following the vendor’s protocol. The seed sequence for rat BAIAP3 is 5′-GACCGTCCGGTGTCACTAC-3′; the nontargeting sequence is 5′-CAACAAGATGAAGAGCACCAA-3′.

### Lentiviral packaging and stable cell generation

20 µg of lentiviral construct, 6 µg of envelop plasmid (pMD2.G; cat. no.12259; Addgene), and 15 µg of packaging plasmid (psPAX2; cat. no.12260; Addgene) were cotransfected into a 10-cm dish of preseeded HEK293FT cells by calcium phosphate transfection. The medium was replaced twice with viral production medium (30% FBS) 6–8 h and 24 h later. Cell debris and intact cells were pelleted at 1,000 *g* for 5 min and filtered out (0.45 µm) from conditioned culture medium after 60 h. Viral particles were pelleted by ultracentrifugation at 33,000 rpm for 2 h with a Ti-70 rotor (Beckman Coulter) and resuspended in 100 µl of cold PBS. Concentrated virus was used to transduce BON or INS-1 cells in the presence of 10 µg/ml protamine sulfate (cat. no. 4020; Sigma).

To generate stable cells for the NPY-Venus secretion assay, BON cells were successively transduced with NPY-Venus and TfR-mCherry lentivirus and diluted into 96-well plates. Single cell–derived colonies were expanded and tested for Ca^2+^-stimulated exocytosis. Clone H8 showed robust secretion and was used for the high throughput screen. TfR-mCherry did not undergo stimulated secretion and was used as a quality control parameter. BAIAP3-HA–expressing cells were generated using a similar procedure and used as a pool without clonal selection.

BAIAP3 knockdown INS-1 cells were generated by transducing an shRNA-encoding lentivirus. A nontargeting lentivirus was used as the control. Successfully transduced cells were selected with 0.4 µg/ml puromycin treatment.

### NPY-Venus secretion assay

BON Clone H8 cells were seeded in 96-well plates at 1.8 × 10^4^ cells/well and incubated for 48 h. For acute stimulation, cells were washed once with 200 µl PSS-Na (145 mM NaCl, 5.6 mM KCl, 2.2 mM CaCl_2_, 0.5 mM MgCl_2_, 15 mM Hepes, and 5.6 mM glucose) after removing culture medium and incubated in 100 µl PSS-Na/ionomycin (1.25 µM) or PSS-Na/DMSO (same concentration as ionomycin) for 10 min at 37°C. The stimulation buffer was transferred to a black-bottom plate. Cells were lysed with 100 µl PSS-Na/1% Triton X-100, and the cell lysate was transferred to a separate black-bottom plate. The fluorescence of NPY-Venus and TfR-mCherry of both stimulation buffer and cell lysates was determined by a Safire II plate reader (Tecan). Background fluorescence of the buffer was subtracted from the samples. The ratio of NPY-Venus in stimulation buffer and total (stimulation buffer + cell lysate) was used to calculate percent secretion:

$$\begin{array}{l} \%\;secretion=\\ \frac{NPY-Venus\;in\;buffer}{NPY-Venus\;in\;buffer+NPY-Venus\;in\;cell\;lysate}\;\times \;100\%. \end{array}$$

For spontaneous secretion, BON clone H8 cells were seeded in 96-well plates and incubated for 2 d. Culture medium was collected after siRNA treatment, and cells were lysed in the same volume of 1% Triton X-100 in PBS. Then, samples were mixed with loading buffer without boiling and loaded onto SDS-PAGE gels for separation. NPY-Venus was quantified by the fluorescence of Venus under AutoQuant software (GE). PC1 was quantified by quantitative Western blotting. Cell number was determined by counting nuclei after Hoechst 33342 staining before lysing cells. An extended version of the protocol for the NPY-Venus and serotonin secretion assays can be found in Bio-protocol (Zhang and Martin, 2018).

### siRNA and plasmid transfection

siRNAs were delivered into BON cells by reverse transfection. In brief, siRNA (50 nM final) was mixed with Metafectamine SI (Biontex Laboratories GmbH) or RNAiMAX (Life Technologies) reagent and transfection buffer in 96-well plate wells, and the cell suspension was added (1.8 × 10^4^ cells per well). Plasmid was transfected into BON cells by Lipofectamine 2000 reagent (Life Technologies) following the vendor’s protocol. 2.5 µg of total plasmid was used for each transfection in 6-well plates or 0.5 µg of total plasmid for 24-well plates. Cells were imaged 24 h after transfection.

### High throughput siRNA screen

siRNAs for C2 domain–containing proteins were selected from the Dharmacon human siGENOME SMARTpool library (GE) and were delivered into cells by Metafectamine SI reagent (Biontex Laboratories GmbH) in 96-well plates. An ionomycin-stimulated NPY-Venus secretion assay was performed 48 h after transfection. The BON NPY-Venus cells (Clone H8) also express a nonsecretable protein, TfR-mCherry. Wells that showed abnormally high TfR-mCherry were excluded from analysis. Nontargeting siRNAs were added to the plate at random positions. Z score was calculated based on the percent secretion of NPY-Venus for each well compared with nontargeting siRNAs. The screen was conducted on three separated occasions, and the mean z score for each gene was used to select hits. The threshold was set as z score = ±2. Cell number was determined by counting nuclei after Hoechst 33342 staining in a separate experiment. The equation to calculate z score was

$$Z\;score=\frac{Y_{siRNA}-Y_{-}}{SD_{-}}.$$

Y_siRNA_ stands for secretion of sample siRNA-treated cells. Y_ and SD_ stand for mean and SD of secretion from control wells.

### Generation of esiRNA

esiRNA is composed of hundreds of different RNA duplexes with each one comprising only a minor fraction; therefore esiRNAs trigger efficient gene silencing with minimal off-target effect (Kittler et al., 2007). esiRNAs were generated following a previously described protocol (Kittler et al., 2005). In brief, two target regions in the BAIAP3 coding sequence were chosen based on RiDDLE prediction and amplified by PCR. Double-strand RNA of target regions (nucleotides 578–1,047 and 2,516–3,035) were generated by an in vitro transcription assay and treated with Dicer to generate esiRNA. esiRNA was purified by Q-Sepharose, and the quality of final product was verified on a 4% agarose gel.

### siRNA rescue studies

BON cells stably expressing NPY-Venus were transduced with lentivirus encoding BAIAP3-myc or BAIAP3-myc–rescue. The rescue construct contains six silent mutations for siRNA no. 3 (Fig. 1 F): 5′-GAG/tcCGTC/gCGT/gTGC/tCAT/cTAC-3′ (underlined nucleotides before the slash are WT, and the nucleotides in lowercase letters after the slash are rescue construct). The resistance of the rescue construct to siRNA knockdown was confirmed by Western blotting. Two cell populations expressing BAIAP3-myc (in ∼49.6% of cells) and BAIAP3-myc–rescue (in ∼43.3% of cells) at similar levels were used for the NPY-Venus secretion assay following the same protocols described in the siRNA and plasmid transfection section, except that the final siRNA concentration was 12.5 nM.

### Serotonin secretion assay

BON cells were transfected with siRNA by reverse transfection in 96-well plates. The next day, 0.2 µC [^3^H]serotonin (Perkin Elmer & Analytical Sciences) was added to each well. Th secretion was assayed after 18 h. In brief, cells were washed with 200 µl PSS-Na once after removing culture medium and incubated in 100 µl PSS-Na in the presence of 1.25 µM ionomycin or DMSO (same concentration) for 10 min at 37°C. After collecting the stimulation buffer, cells were lysed with 1% Triton X-100 in PSS-Na. The radioactivity of both stimulation buffer and cell lysates were subsequently determined by a liquid scintillation counter. The ratio of [^3^H]serotonin in stimulation buffer and total (stimulation buffer + cell lysate) was calculated to indicate the percent secretion. Because of residual [^3^H]serotonin remaining after removal of the labeling medium, the background (DMSO-treated sample) was high and subtracted to calculate the Ca^2+^-dependent secretion.

### Insulin secretion assay

Stable INS-1 cells were generated after transducing cells with a *BAIAP3* shRNA lentivirus (seed sequence 5′-GACCGTCCGGTGTCACTAC-3′). Control cells were transduced with a control shRNA lentivirus (seed sequence 5′-CAACAAGATGAAGAGCACCAA-3′). Control and BAIAP3 knockdown INS-1 cells were seeded into 96-well plates at a density of 10^5^ cells per well. After 2 d of culturing, cells were re-fed with fresh culture medium. The next day, cells were washed twice with Krebs buffer (118 mM NaCl, 4.7 mM KCl, 1.2 mM MgSO_4_, 1.2 mM KH_2_PO_4_, 25 mM NaHCO_2_, 5 mM Hepes, 2.5 mM CaCl_2_ supplemented with an additional 15 mM Hepes [final 20 mM], fatty acid–free BSA [0.2%], and1.5 mM glucose) and preincubated at 37°C for 2 h with the same washing solution. INS-1 cells were stimulated with 1.5, 7, and 15 mM glucose in the same solution for 2 h. Supernatant was collected, and insulin concentration was determined by an in-house ELISA assay. The cell number of each well was determined by measuring DNA content of cell lysates after the secretion assay with a PicoGreen dsDNA Assay kit (Life Technologies). The Krebs buffer was warmed to 37°C and gassed with 95% O_2_/5% CO_2_ to normalize the pH before the experiment.

### Fluorescence microscopy imaging and image analysis

For plasmid transfection, BON cells were plated on poly-d-lysine–coated coverslips and transfected with Lipofectamine 2000 reagent (Life Technologies) according to the manufacturer’s recommendation. For siRNA transfection, the reaction was done in silicone compartments (flexiPERM; Sarstedt Inc.) attached by a poly-d-lysine–coated coverslip. Cells were fixed in 4% formaldehyde (8 min) and permeabilized with 0.1% Triton X-100 in PBS with 5% BSA (10 min). After blocking with 5% BSA in PBS (30 min), primary antibodies were incubated overnight at 4°C and stained with secondary antibodies for 1 h. Coverslips were mounted with SlowFade reagent (Life Technologies) and sealed with nail polish. Samples were imaged with either a confocal microscope (A1R^+^; Nikon) equipped with GaAsp detectors and an oil objective (60×/NA1.40; Plan Apo) or a STORM/TIRF microscope (Nikon) equipped with a laser (MLC400B; Agilent), charge-coupled device camera (iXon Ultra; Andor), and an oil objective (100×/NA1.49; ApoTIRF). The microscopes were controlled by Nikon Elements software. Image acquisition was performed at ambient temperature. GFP, Venus, and mCherry were used for live-cell studies. Secondary antibodies were labeled with Alexa Fluor 488, 568, or 647 or DyLight 405. Nuclei were stained with Hoechst 33342 (cat. no. 1399; Life Technologies). Images were analyzed with ImageJ (National Institutes of Health) or Nikon Elements software.

BAIAP3 colocalization with Rabs and CgA was analyzed by the colocalization module of Nikon Elements. Pearson’s correlation coefficient was used to indicate the colocalization level. CgA and VAMP4 colocalization was performed using the ImageJ plugin JACoP (Just Another Colocalization Plugin).

To count DCVs that reached the TIRF footprint of BON cells, live-cell images of randomly selected cells were captured with a STORM/TIRF microscope. DCVs were determined by the Find maxima module of ImageJ using the same threshold for all images. Source code is deposited at Github (https://github.com/kingmanzhang/ImageJ_macro_microscopy; vesicle counting under TIRF.txt).

For the BAIAP3-mCherry recruitment assay, doubly transfected (BAIAP3-mCherry and GFP-VAMP4) BON cells were stimulated with 2.5 µM ionomycin in the presence of 2.2 mM external Ca^2+^. Every 10 s, a dual-channel image of the equatorial section of the cells was captured by a confocal microscope (A1; Nikon). To determine the fluorescence of BAIAP3-mCherry on the Golgi, on the plasma membrane, and in the cytoplasm, CellProfilers was used to create masks for those regions. The GFP channel was used to create a mask for the TGN region. Cell boundary and the nucleus were automatically detected by CellProfiler. Regions within five pixels to cell boundary were regarded as the plasma membrane. Cytoplasmic regions were calculated by subtracting the nucleus, Golgi, and plasma membrane regions from the entire cell. Fluorescent percent changes (ΔF divided by F_0_) were reported.

### Electron microscopy

Cells on coverslips were fixed by 2.5% glutaraldehyde and 2% paraformaldehyde and postfixed by 1% osmium tetroxide and 1% potassium ferricyanide. After fixation, cells were stained with saturated aqueous uranyl acetate and dehydrated in ethanol and propylene oxide. Cells were infiltrated and embedded in Durcupan ACM resin. Cell sections were imaged by a transmission electron microscope (CM120; Philips). The number and size of DCVs in EM micrographs was determined with the Particle Analysis plugin under ImageJ.

### Mass spectrometry

BON cells were plated on nine 10-cm dishes. The next day, three plates were replaced with serum-free culture medium (5 ml per plate) after five extensive washes with PBS. After 22 h, serum-free culture medium that contained spontaneously secreted protein was collected from the three plates. For the other six plates, cells were washed extensively and incubated in PSS-Na (5 ml per plate) in the presence of 1.25 µM ionomycin or DMSO (same concentration) for 10 min at 37°C. All samples were centrifuged to remove cell debris, protease inhibitor (Roche) was added, and samples were snap frozen. Samples were separated into protein and peptide fractions by a 10,000-Dalton molecular weight cutoff centrifugal filter (EMD Millipore), and the protein fraction was digested with trypsin. Samples were separated by liquid chromatography and analyzed by tandem mass spectrometry using a Q-Exactive Orbitrap mass spectrometer (Thermo Fisher Scientific). Relative protein abundance was determined by label-free quantification using the median of area under the curve of MS1 peaks (Cox and Mann, 2008; Cox et al., 2011).

### INS-1 fractionation

INS-1 cells harvested from five dishes (15 cm) were resuspended in 2 ml KGlu-EGTA (20 mM Hepes, pH 7.1, 120 mM potassium glutamate, 20 mM potassium acetate, and 2 mM EGTA). Cells were passed through a ball-bearing cell homogenizer (4-µm clearance) 18 times. Nuclei and unbroken cells were pelleted by low speed centrifugation at 1,000 *g* for 10 min. The postnuclear supernatant was subjected to sucrose gradient (0.27–2 M, total volume 11 ml) separation at 114,000 *g* for 18 h. The gradient was separated into 12 fractions (1 ml each). Proteins in each fraction were concentrated by TCA precipitation and analyzed by Western blotting.

### GST pulldown assay

Constructs for GST-tagged VAMP3 (1-75), STX6 (1-230), STX16 (1-296), and Vti1A (1-192) were a gift from S. Pfeffer (Stanford University, Stanford, CA). Construct for GST-tagged VAMP4 (1-114) was a gift from J. Bonifacino (National Institutes of Health, Bethesda, MD). Proteins were purified from *Escherichia coli* as previously described (Ganley et al., 2008). For binding assays, 20 µg GST-SNAREs were immobilized on 10 µl BSA (5%)-blocked glutathione sepharose beads and incubated in the presence of 100 µM calcium or EGTA/EDTA (0.5 mM each) with 500 µg cell lysate from BON cells stably expressing BAIAP3-HA. After an overnight incubation at 4°C, beads were washed three times, and proteins were eluted with 20 mM glutathione for analysis by Western blotting. 

### Lipid blot assay

Recombinant His-MBP-BAIAP3 was purified from HEK293FT cell lysates after transfection with pcDNA3-His-MBP-BAIAP3 using Ni-NTA beads. After blocking the lipid blot for 1 h with 5% BSA, PIP strips (Echelon) were incubated with 0.5 µg/ml His-MBP-BAIAP3 in the presence of 10 µM Ca^2+^ or 50 µM EGTA for 1 h. After washing three times, bound BAIAP3 was determined with an MBP antibody (cat. no. E8032; New England Biolabs) and HRP-conjugated secondary antibody. His-MBP was used as a control. Three independent experiments were conducted.

### Statistical analysis

All statistical analysis was conducted in R software. Significance was determined using Student’s *t* test, unless indicated otherwise. Bar graphs are presented as mean ± SD unless otherwise stated. *, P < 0.05; **, P < 0.01. Figures were generated with Prism (GraphPad) or ggplot2 in R.

### Online supplemental material

Fig. S1 shows the lack of Munc13-4 and VAMP2 in BON cells, selected result from the siRNA screen, and BAIAP3 knockdown phenotype by esiRNAs. Fig. S2 shows the identification of CgA-derived peptides from acutely stimulated and resting BON cells. Fig. S3 shows DCV size distribution in BON cells and insulin granule number and size changes in INS-1 cells. Fig. S4 shows the immunofluorescent staining of endogenous BAIAP3 in INS-1 cells. Fig. S5 shows the lipid binding specificity of BAIAP3 in a protein-lipid overlay assay. Fig. S6 shows the conservation of Ca^2+^-binding residues in the C2 domains of BAIAP3. Table S1 lists all human C2 domain–containing proteins in the siRNA screen. Table S2 lists all hits identified from the siRNA screen. Video, related to Fig. 6 F, shows Ca^2+^-stimulated recruitment of BAIAP3 to the Golgi and plasma membrane in BON cells.

## Supplementary Material

Supplemental Materials (PDF)

Video 1
