# Supplementary material for: BAIAP3, a C2 domain–containing Munc13 protein, controls the fate of dense-core vesicles in neuroendocrine cells
Source: J Cell Biol. 2017 Jul 3;216(7):2151–66. doi: 10.1083/jcb.201702099 (PMC5496627; doi:10.1083/jcb.201702099)
Supplement: Supplemental Materials (PDF) [file JCB_201702099_sm.pdf]

Zhang et al., https://doi.org/10.1083/jcb.201702099

THE JOURNAL OF CELL BIOLOGY

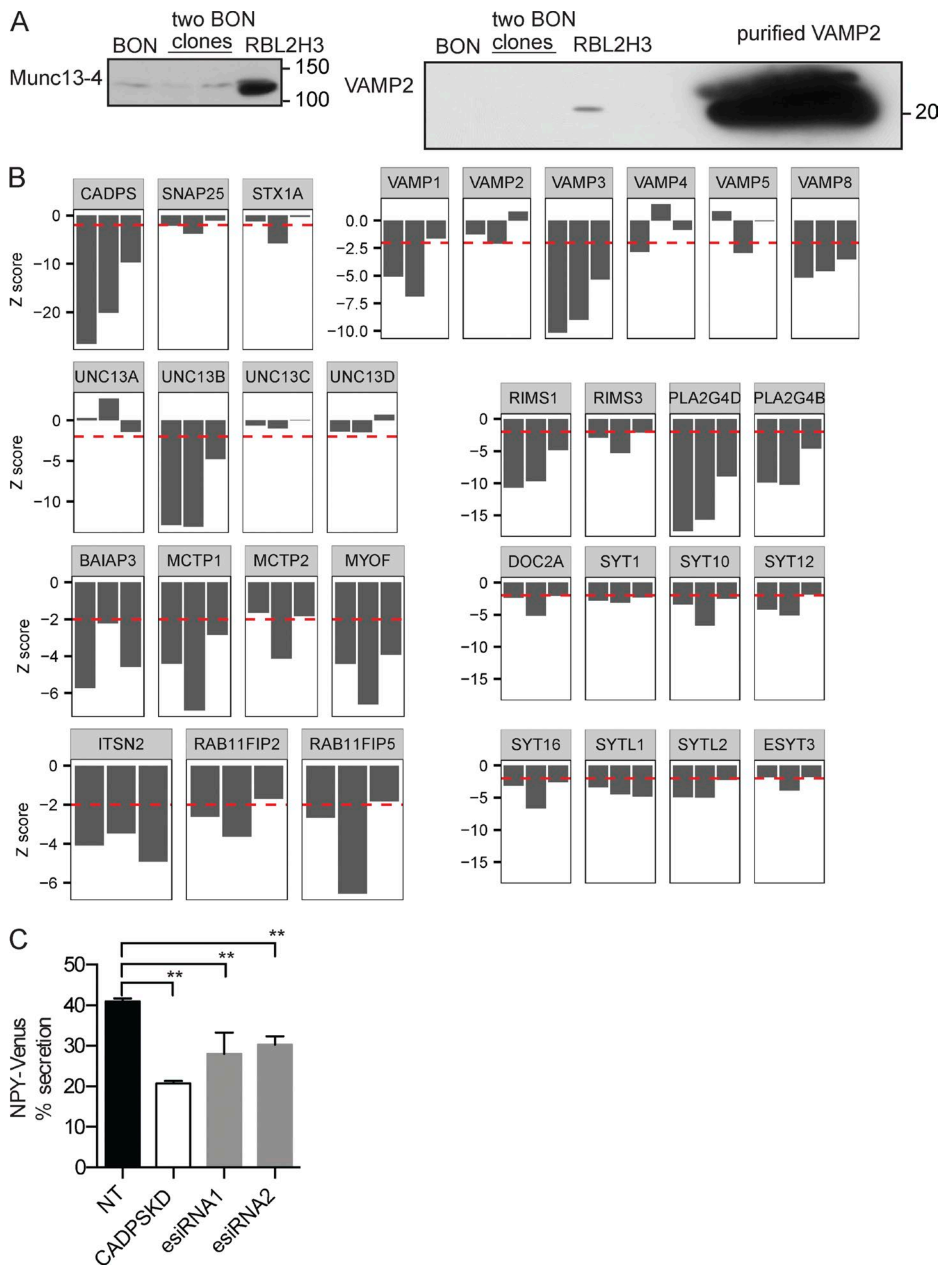

Figure S1. **High throughput screen of C2 domain–containing proteins (related to Fig. 1).** (A) Munc13-4 and VAMP2 are not detected in BON cells. Parental BON cells and two single cell–derived clones were tested and compared with rat basophilic leukemia (RBL) cells that express abundant Munc13-4. Molecular mass is shown in kilodaltons. (B) Z scores for selected genes of C2 domain–containing proteins. Each bar represents a z score from one experiment. The screen was performed three times. Red dashed lines indicate the threshold for hit identification (z score = −2). (C) Validation of BAIAP3 knockdown (KD) effect on NPY-Venus percent secretion with the two esiRNA pools targeting regions shown in Fig. 1 D. *n* = 5. Data are expressed as mean ± SD. P-values were obtained by a two-tailed Student's *t* test. **, P < 0.01. NT, nontargeting.

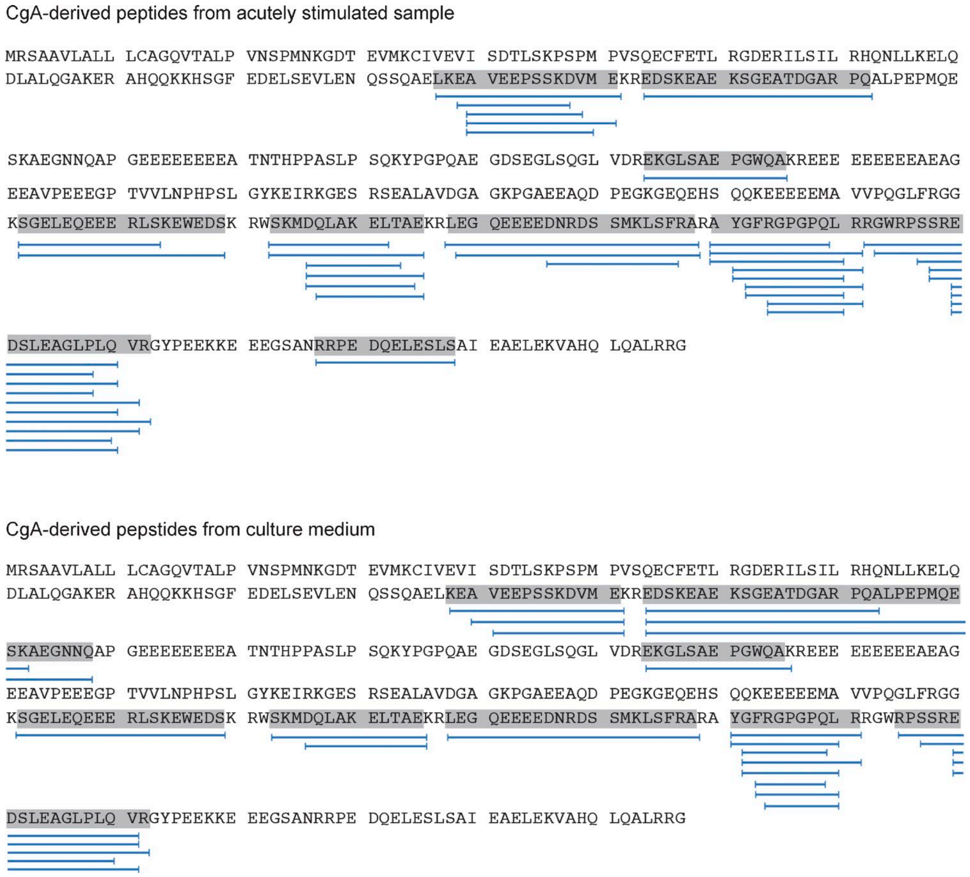

Figure S2. **Identification of CgA-derived peptides from the acutely stimulated and resting BON cells (related to Fig. 2).** The samples were passed through a 10,000-Dalton molecular weight cutoff centrifugal filter, and peptides in the flow through were determined. Only peptides aligned to CgA are shown. Blue ranges indicate peptides identified by mass spectrometry. Gray highlighting indicates overall sequence coverage by peptide identification.

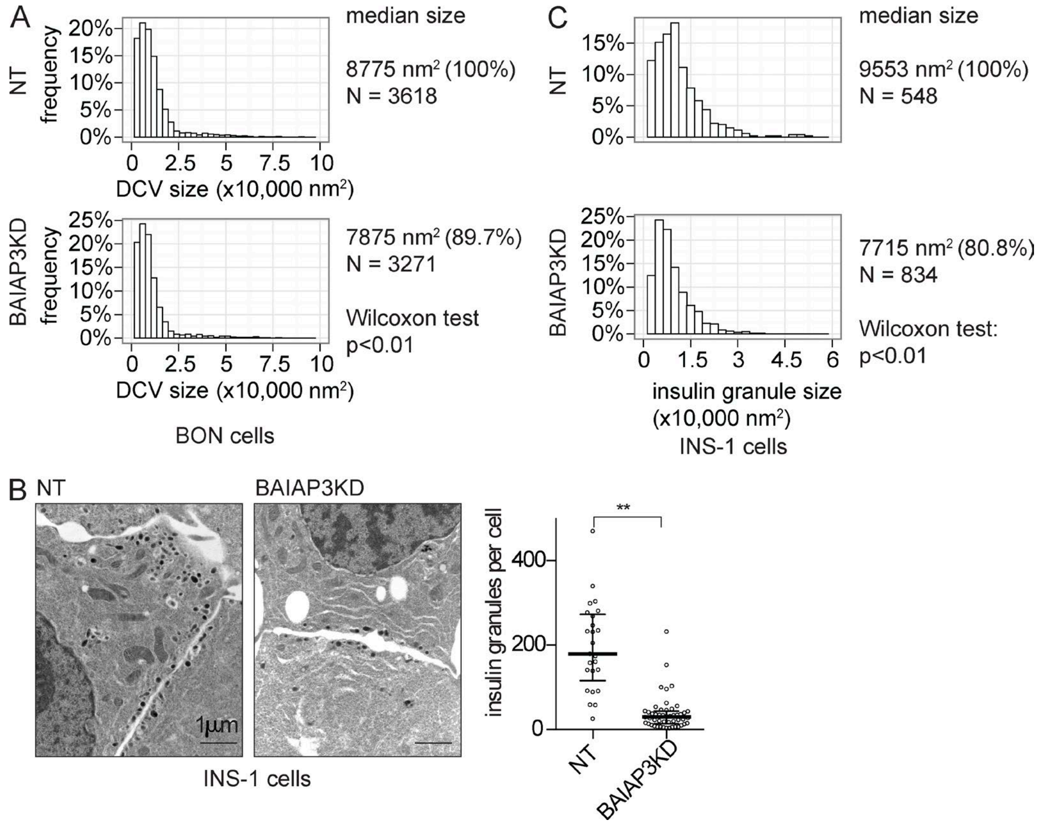

Figure S3. **Electron microscopic analysis of DCVs after BAIAP3 knockdown in BON and INS-1 cells (related to Figs. 3 and 4).** (A) DCV size distribution in control and BAIAP3 knockdown (BAIAP3KD) BON cells. NT, nontargeting. (B) Loss of insulin granules after BAIAP3 knockdown in INS-1 cells. *n* = 25 nontargeting and 49 BAIAP3 knockdown cells. Data are represented as median ± quantile. P-value was obtained by Wilcoxon test. **, P < 0.01. (C) Insulin granule size distribution in control and BAIAP3 knockdown INS-1 cells.

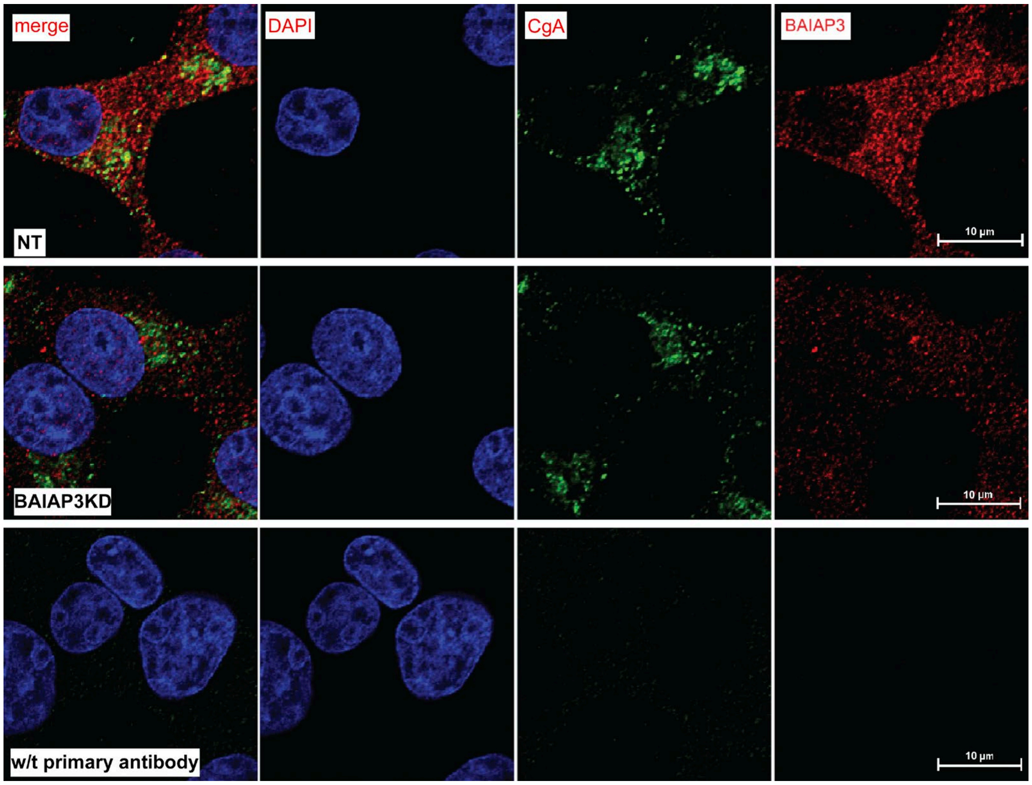

Figure S4. **Endogenous BAIAP3 localization in INS-1 cells (related to Fig. 5).** Stable INS-1 cells transduced with nontargeting (NT)-shRNA or BAIAP3-shRNA were immunostained with a BAIAP3 antibody and with CgA antibody to detect insulin granules. BAIAP3 knockdown (BAIAP3KD) was verified by Western blotting and shown in Fig. 4 B. Bars, 10 µm. w/t, without.

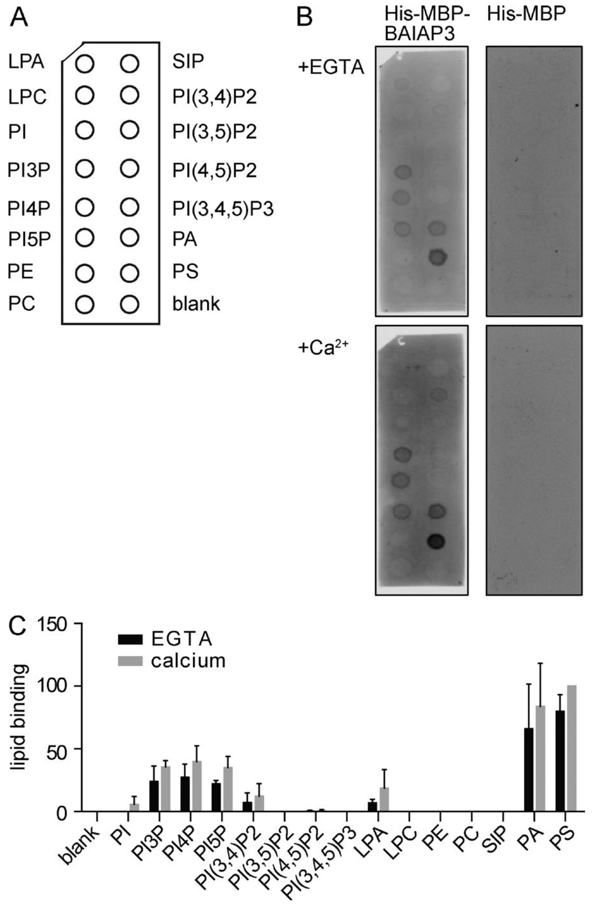

Figure S5. **BAIAP3-lipid binding specificity (related to Fig. 6).** (A) The composition of the lipid blot. LPA, lysophosphatidic acid; LPC, lysophosphocholine; PA, phosphatidic acid; PC, phosphatidylcholine; PE, phosphatidylethanolamine; PI, phosphatidylinositol; PI3P, PI (3) phosphate; PI4P, PI (4) phosphate; PI5P, PI (5) phosphate; PI(3,4)P2, PI (3,4) bisphosphate; PI(3,5)P2, PI (3,5) bisphosphate; PI(3,4,5)P3, PI (3,4,5) trisphosphate; PI(4,5)P2, PI (4,5) bisphosphate; PS, phosphatidylserine; S1P, sphingosine 1-phosphate. (B) A representative result from the protein-lipid overlay assay using recombinant His-MBP-BAIAP3 or His-MBP. (C) Quantification of bound BAIAP3 by different lipids. $n = 3$. Although there was a trend toward Ca^{2+} increasing lipid binding, this was not statistically significant by Student's *t* test. Data are mean ± SD.

```
BAIAP3C2A             1 E-A-IERVRKAKAPT---------------YALKVSVMRAKNLLAKDPNGFSDPYCMLGI
BAIAP3C2B             1 --GRLSVRCHYEAAE---------------QRLAVEVLHAADLLPLDANGLSDPFVIVEL
Munc13-1C2B           1 ------------------------------AKISITVVCAQGLQAKDKTGSSDPYVTVQV
Munc13-2C2B           1 ------------------------------AKITITVVCAQGLQAKDKTGSSDPYVTVQV
Syt1C2A               1 L-GKLQYSLDYDFQN---------------NQLLVGIIQAAELPALDMGGTSDPYVKVFL
Syt1C2B               1 L-GDICFSLRYVPTA---------------GKLTVVILEAKNLKKMDVGGLSDPYVKIHL
Munc13-4C2A           1 Y-L-QEAF-HVEPEEHQQTLQRVRELEKPIFCLKATVKQAKGILGKDVSGFSDPYCLLGI
Munc13-4C2B           1 ELGAVTVKASYRASE---------------QKLRVELLSASSLLPLDSNGSSDPFVQLTL
Rabphilin3aC2A        1 L-GALEFSLLYDQDN---------------SSLQCTIIKAKGLKPMDSNGLADPYVKLHL
Rabphilin3aC2B        1 ---KILVSLMYSTQQ---------------GGLIVGIIRCVHLAAMDANGYSDPFVKLWL
UVRAGC2               1 ---------------------------------HLRNIAARNIVNRNGHQLLDTYFTLHL
PRKCAC2               1 R-GRI--YLKAEVAD---------------EKLHVTVRDAKNLIPMDPNGLSDPYVKLKL
                                                              *     *

BAIAP3C2A            44 LPASDATR-EPRAQKEQRFGFRKGSKRGGPLPAKCIQVTEVKSSTLNPVWKEHFLFE-IE
BAIAP3C2B            44 GPPHL-------------------------FPLVRSQRTQVKTRTLHPVYDELFYFS-VP
Munc13-1C2B          31 GK--------------------------------TKKRTKTIYGNLNPVWEENFHFE-CH
Munc13-2C2B          31 SK--------------------------------TKKRTKTIFGNLNPVWEEKFHFE-CH
Syt1C2A              45 LPD-----------------------------KKKKFETKVHRKTLNPVFNEQFTFK-VP
Syt1C2B              45 MQNGK---------------------------RLKKKKTTIKKNTLNPYYNESFSFE-VP
Munc13-4C2A          58 EQGVGVPGGSPGSRHRQKAVV------RHTIPEEETHRTQVITQTLNPVWDETFILE-FE
Munc13-4C2B          46 EPRHE-------------------------FPELAARETQKHKKDLHPLFDETFEFL-VP
Rabphilin3aC2A       45 LPGAS---------------------------KSNKLRTKTLRNTRNPIWNETLVYHGIT
Rabphilin3aC2B       43 KPDM---------------------------GKKAKHKTQIKKKTLNPEFNEEFFYD-IK
UVRAGC2              28 CSTEK-------------------------I-YKEFYRSEVIKNSLNPTWRS-LDFG-IM
PRKCAC2              43 IPDPK---------------------------NESKQKTKTIRSTLNPQWNESFTFK-LK

BAIAP3C2A           102 D--V--STDQLHLDIWDHDDDVSL-VEAC-------------------RKLNEVIGLKG-
BAIAP3C2B            78 AEACRRRAACVLFTVMDHDWLS----------------------------TNDFAGEAA-
Munc13-1C2B          58 N-----SSDRIKVRVWDEDDDIKS-RVKQ----------------RFKRESDDFLGQTII
Munc13-2C2B          58 N-----SSDRIKVRVWDEDDDIKS-RVKQ----------------RLKRESDDFLGQTII
Syt1C2A              75 YSEL--GGKTLVMAVYDFDRF----------------------------SKHDIIGEFKV
Syt1C2B              77 FEQ--IQKVQVVVTVLDYDKIG----------------------------KNDAIGKVFV
Munc13-4C2A         111 D--I--TNASFHLDMWDLDTVESV-RQKLGELTDLHGLRRIFKEARKDKGQDDFLGNVVL
Munc13-4C2B          80 AEPCRKAGACLLLTVLDYDTLG----------------------------ADDLEGEAFL
Rabphilin3aC2A       78 DEDMQR--KTLRISVCDEDKFG----------------------------HNEFIGETRF
Rabphilin3aC2B       75 HSDL--AKKSLDISVWDYDIG----------------------------KSNDYIGGCQL
UVRAGC2              60 PDRLDTSVSCFVVKIWGGKENIYQLLIEWK----------------VCLDGLKYLGQQIH
PRKCAC2              75 PSDK-D--RRLSVEIWDWDRTT----------------------------RNDFMGSLSF
                                        * *                               *

BAIAP3C2A               ------------------------------------
BAIAP3C2B               ------------------------------------
Munc13-1C2B          96 EVRTL--SGEM-D-VWYNLDKRTDKSAVSGAIRLHI
Munc13-2C2B          96 EVRTL--SGEM-D-VWYNLEKRTDKSAVSGAIRLQI
Syt1C2A             105 PMNTVDFGHVT-E-EWRDLQ---------------S
Syt1C2B             107 GYNST--GAEL-R-HWSDMLANPRRP-IAQWHTLQ-
Munc13-4C2A         166 RLQDL--RCRE-D-QWYP-----------------L
Munc13-4C2B         112 PLREV--PGLSGSEEPGEVP--------------Q-
Rabphilin3aC2A      108 SLKKL--KPNQ-R-KNFNIC--------------L-
Rabphilin3aC2B      105 GISAK--GERL-K-HWYECLKNKDKK-IERWHQ--L
UVRAGC2             104 -ARN-----QN-E-IIFGL--------NDGY-----
PRKCAC2             104 GVSEL--MKMPAS-GWYKLLNQEEGE----YYNVP-
```

*
predicted Ca2+-binding sites

Figure S6. **Conservation of Ca^{2+}-binding residues in the C2 domains of BAIAP3 (related to Fig. 6).** Negatively charged residues that are predicted Ca^{2+}-binding residues are labeled with an asterisk. Black highlighting indicates amino acids with identity >50%; gray highlighting indicates amino acids with similarity >50%.

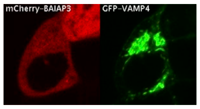

Video 1. **Ca^{2+}-stimulated recruitment of BAIAP3 to the Golgi and plasma membrane in BON cells (related to Fig. 6 F).** Red, mCherry-BAIAP3; green, GFP-VAMP4 labeling Golgi. Intracellular Ca^{2+} was raised at the third frame with 2.5 µM ionomycin. An image of the equatorial section of the cell was captured every 10 s by a confocal microscope. The video is displayed at one frame per second.

Table S1. **List of human C2 domain–containing proteins for siRNA screen**

UniParc	Gene Name	Gene ID
Q9UBL6	*CPNE7*	27132
P47712	*PLA2G4A*	5321
P0C869	*PLA2G4B*	100137049
Q86XP0	*PLA2G4D*	283748
Q3MJ16	*PLA2G4E*	123745
Q68DD2	*PLA2G4F*	255189
Q9NQ66	*PLCB1*	23236
Q00722	*PLCB2*	5330
Q01970	*PLCB3*	5331
Q15147	*PLCB4*	5332
P51178	*PLCD1*	5333
Q8N3E9	*PLCD3*	113026
Q9BRC7	*PLCD4*	84812
Q9P212	*PLCE1*	51196
P19174	*PLCG1*	5335
P16885	*PLCG2*	5336
Q4KWH8	*PLCH1*	23007
O75038	*PLCH2*	9651
Q15111	*PLCL1*	5334
Q9UPR0	*PLCL2*	23228
Q86YW0	*PLCZ1*	89869
P20936	*RASA1*	5921
Q15283	*RASA2*	5922
Q14644	*RASA3*	22821
O43374	*RASA4*	10156
O95294	*RASAL1*	8437
Q96PV0	*SYNGAP1*	8831
P21579	*SYT1*	6857
Q9P2Y5	*UVRAG*	7405
Q9UJF2	*RASAL2*	9462
Q8IV01	*SYT12*	91683
Q12979	*ABR*	29
O94812	*BAIAP3*	8938
Q9ULU8	*CADPS*	8618
Q86UW7	*CADPS2*	93664
Q96A23	*CPNE4*	131034
Q9HCH3	*CPNE5*	57699
O95741	*CPNE6*	9362
Q86YQ8	*CPNE8*	144402
Q5VWQ8	*DAB2IP*	153090
Q14184	*DOC2B*	8447
O75923	*DYSF*	8291
Q15811	*ITSN1*	6453
Q9NZM3	*ITSN2*	50618
Q86YS7	*C2CD5*	9847
Q9HC10	*OTOF*	9381
Q86UR5	*RIMS1*	22999
Q9UQ26	*RIMS2*	9699
Q9H426	*RIMS4*	140730
Q9Y2J0	*RPH3A*	22895
Q9BT88	*SYT11*	23208
Q9BQS2	*SYT15*	83849
Q9H2B2	*SYT4*	6860
O00445	*SYT5*	6861
Q5T7P8	*SYT6*	148281
Q86SS6	*SYT9*	143425
Q8IYJ3	*SYTL1*	84958
Q9HCH5	*SYTL2*	54843
Q8N9U0	*TC2N*	123036
Q9H0E2	*TOLLIP*	54472
Q9UPW8	*UNC13A*	23025
Q70J99	*UNC13D*	201294
O14795	*UNC13B*	10497
Q4AC94	*C2CD3*	26005
Q9P2K1	*CC2D2A*	57545
Q99829	*CPNE1*	8904
Q96FN4	*CPNE2*	221184
Q14183	*DOC2A*	8448
Q9NZM1	*MYOF*	26509
Q6WKZ4	*RAB11FIP1*	80223
Q9BXF6	*RAB11FIP5*	26056
Q9UJD0	*RIMS3*	9783
Q96KN7	*RPGRIP1*	57096
Q6XYQ8	*SYT10*	341359
Q7L8C5	*SYT13*	57586
Q8NB59	*SYT14*	255928
Q8N9I0	*SYT2*	127833
Q9BQG1	*SYT3*	84258
O43581	*SYT7*	9066
Q8NBV8	*SYT8*	90019
Q8TDW5	*SYTL5*	94122
Q8NB66	*UNC13C*	440279
Q9ULE0	*WWC3*	55841
Q86YV0	*RASAL3*	64926
Q8NCU7	*C2CD4A*	145741
A6NLJ0	*C2CD4B*	388125
Q6P1N0	*CC2D1A*	54862
Q5T0F9	*CC2D1B*	200014
Q8N5R6	*CCDC33*	80125
Q8IYJ1	*CPNE9*	151835
Q9BSJ8	*ESYT1*	23344
A0FGR8	*ESYT2*	57488
A0FGR9	*ESYT3*	83850
A0AVI2	*FER1L5*	90342
Q2WGJ9	*FER1L6*	654463
Q6DN14	*MCTP1*	79772
Q6DN12	*MCTP2*	55784
Q7L804	*RAB11FIP2*	22841
Q68CZ1	*RPGRIP1L*	23322
Q58G82	*SYT14P1*	401135
Q17RD7	*SYT16*	83851
Q9BSW7	*SYT17*	51760
Q8IX03	*WWC1*	23286
Q6AWC2	*WWC2*	80014
P11274	*BCR*	613
O75131	*CPNE3*	8895
O00443	*PIK3C2A*	5286
O00750	*PIK3C2B*	5287
O75747	*PIK3C2G*	5288
Q16512	*PKN1*	5585
Q16513	*PKN2*	5586
P17252	*PRKCA*	5578
P05771	*PRKCB*	5579
Q05655	*PRKCD*	5580
Q02156	*PRKCE*	5581
P05129	*PRKCG*	5582
P24723	*PRKCH*	5583
Q04759	*PRKCQ*	5588
Q96PE3	*INPP4A*	3631
O15327	*INPP4B*	8821
O15484	*CAPN5*	726
Q9Y6Q1	*CAPN6*	827

Table S1. **List of human C2 domain–containing proteins for siRNA screen** *(Continued)*

UniParc	Gene Name	Gene ID	UniParc	Gene Name	Gene ID
Q76N89	*HECW1*	23072	O00308	*WWP2*	11060
Q9P2P5	*HECW2*	57520	Q4VX76	*SYTL3*	94120
Q96J02	*ITCH*	83737	Q96C24	*SYTL4*	94121
Q96PU5	*NEDD4L*	23327	Q8TF44	*C2CD4C*	126567
Q9HCE7	*SMURF1*	57154	B7Z1M9	*C2CD4D*	100191040
Q9HAU4	*SMURF2*	64750	A9Z1Z3	*FER1L4*	80307
Q9H0M0	*WWP1*	11059	Q9Y6V0	*PCLO*	27445

Table S2. **List of candidate hits of C2 domain–containing proteins**

Gene	Protein name	Z score
CADPS	CAPS/calcium-dependent secretion activator	−15.2
PLA2G4D	phospholipase A2 group IVD	−11.8
CADPS	**CAPS/calcium-dependent secretion activator**	**−8.7**
UNC13B	Munc13-2/mammalian Unc13-homolog B	−8.4
RIMS1	RIM/regulating synaptic membrane exocytosis 1	−7.0
PLA2G4B	phospholipase A2 group IVB	−6.9
VAMP3	**VAMP3/vesicle-associated membrane protein 3**	**−6.9**
MYOF	myoferlin	−5.5
MCTP1	multiple C2 and transmembrane protein 1	−5.1
SYT10	synaptotagmin 10	−4.5
SYT16	synaptotagmin 16	−4.3
RPGRIP1	retinitis pigmentosa GTPase regulator interacting protein 1	−4.2
TOLLIP	Toll-interacting protein	−4.2
CPNE4	copine 4	−4.1
PLCB1	phospholipase C β 1	−4.1
SYTL1	synaptotagmin-like protein 1	−4.0
ITSN2	intersectin 2	−3.9
VAMP8	**VAMP8/vesicle-associated membrane protein 8**	**−3.9**
PRKCA	protein kinase C α	−3.8
RAB11FIP5	RAB11 family–interacting protein 5	−3.8
VAMP1	**VAMP1/vesicle-associated membrane protein 1**	**−3.8**
BAIAP3	BAI1-associated protein 3	−3.8
RIMS3	RIM3/regulating synaptic membrane exocytosis 3	−3.7
CAPN6	calpain 6	−3.6
FER1L5	fer-1–like family member 5	−3.6
RASAL	RASAL	−3.5
SYTL2	synaptotagmin-like protein 2	−3.5
DOC2A	double C2 domain α	−3.4
SYT12	synaptotagmin 12	−3.2
FER1L6	fer-1–like family member 6	−3.2
CPNE7	copine 7	−3.2
PLCL1	phospholipase C–like 1	−3.1
CADPS2	calcium-dependent secretion activator 2	−2.9
RAB11-FIP2	RAB11 family–interacting protein 2	−2.9
MCTP2	multiple C2 and transmembrane domain–containing 2	−2.6
ESYT3	extended synaptotagmin 3	−2.6
DYSF	dysferlin	−2.6
SYT1	synaptotagmin 1	−2.5
RASA3	RAS p21 protein activator 3	−2.5
SNAP25	**SNAP25**	**−2.4**
CCDC33	coiled-coil domain–containing 33	−2.2
STX1A	**syntaxin 1A**	**−2.1**
PLCZ1	phospholipase C ζ 1	−2.1
RASA4	RAS p21 protein activator 4	−2.1
ESYT2	extended synaptotagmin 2	−2.0
RASAL2	RAS protein activator–like 2	−2.0

Bold are controls.
